# Dose–response relationship between tea consumption and mortality, cardiovascular, cerebrovascular, and renal outcomes in metabolic dysfunction-associated steatotic liver disease: a prospective cohort study

**DOI:** 10.3389/fnut.2026.1945413

**Published:** 2026-09-24

**Authors:** Tom Ryu, Jaejun Lee, Ji Won Han, Hyun Yang, Keungmo Yang

**Affiliations:** 1 Department of Internal Medicine, Institute for Digestive Research, Digestive Disease Center, Soonchunhyang University College of Medicine, Seoul, Republic of Korea; 2 Department of Internal Medicine, Division of Gastroenterology and Hepatology, College of Medicine, The Catholic University of Korea, Seoul, Republic of Korea

**Keywords:** cardiometabolic outcome, clinical outcomes, dose–response, metabolic dysfunction-associated steatotic liver disease, tea

## Abstract

**Background:**

Tea is a widely consumed plant-derived beverage with potential metabolic and vascular effects, but its prognostic dose–response relationship in metabolic dysfunction-associated steatotic liver disease (MASLD) remains unclear.

**Methods:**

We analyzed 360,548 UK Biobank participants, including 169,241 individuals with MASLD, 146,559 with no steatotic liver disease (SLD), and 44,748 with MASLD and increased alcohol intake (MetALD). Hepatic steatosis was defined using a fatty liver index (FLI) ≥ 60. Tea consumption was categorized as none, 1–2, 3–4, 5–6, and ≥7 cups/day. The outcomes included all-cause mortality, cardiovascular disease, cerebrovascular disease, and chronic kidney disease. Cox regression, restricted cubic spline (RCS) analyses, inverse probability of treatment weighting (IPTW), subgroup analyses, and multiple sensitivity analyses were performed. The no SLD and MetALD cohorts were analyzed as comparators.

**Results:**

Among participants with MASLD, 16,611 deaths, 18,004 cardiovascular disease events, 7,106 cerebrovascular disease events, and 9,132 chronic kidney disease events occurred during follow-up. Tea consumption showed a reproducible non-linear association with mortality and cardiometabolic outcomes in MASLD. The RCS-derived nadirs ranged from 1.6 to 2.5 cups/day across the outcomes. In fully adjusted models, tea intake of 1–2 cups/day was associated with lower all-cause mortality (HR: 0.85, 95% CI: 0.81–0.90; *p* < 0.001) and a lower risk of cerebrovascular disease (HR: 0.83, 95% CI: 0.77–0.90; *p* < 0.001), whereas the ≥7 cups/day category was associated with an increased risk of cardiovascular disease (HR: 1.09, 95% CI: 1.02–1.15; *p* = 0.006).

**Conclusion:**

Tea consumption showed a consistent J-shaped association with mortality and major cardiometabolic outcomes in MASLD, with the lowest estimated risks occurring within a low-to-moderate range of intake. These findings support a dose-aware interpretation of tea consumption in MASLD.

## 1 Introduction

MASLD is the current term for hepatic steatosis occurring in the presence of cardiometabolic risk factors (1). Contemporary guidelines emphasize that MASLD is associated not only with liver-related outcomes but also with cardiovascular disease, chronic kidney disease, and premature mortality (2). Because many patients continue to be managed primarily through lifestyle interventions and cardiometabolic risk modification, lifestyle exposure remains an important clinical priority (3).

Tea is one of the most widely consumed beverages worldwide and occupies a unique position between habitual diet and phytopharmacology (4, 5). Although tea is not administered as a standardized phytopharmaceutical intervention, it represents a widely consumed plant-derived exposure containing multiple bioactive compounds that may be relevant to the pathophysiology of MASLD (6, 7). Green tea is rich in catechins, particularly epigallocatechin-3-gallate, whereas black tea contains theaflavins and thearubigins generated during oxidation; both types also contain caffeine, theobromine, and L-theanine (8). Tea contains multiple bioactive compounds that have been linked to antioxidant, anti-inflammatory, endothelial, and lipid-modulating effects (9).

Clinical and epidemiological evidence on tea, however, remains difficult to translate into dosing advice. Meta-analyses of general population studies suggest that tea consumption is associated with lower cardiovascular and all-cause mortality, and a recent dose–response synthesis reported that the greatest reduction in all-cause mortality occurs approximately 1.5–2.0 cups/day (10, 11). Trials and meta-analyses in non-alcoholic fatty liver disease (NAFLD) have also suggested beneficial effects of green tea or catechins on liver enzymes and fat deposition (12, 13). However, previous NAFLD/MASLD studies have mainly examined disease prevalence or short-term hepatic outcomes rather than non-linear associations between tea consumption and long-term clinical outcomes (14). Therefore, the prognostic dose–response relationship of habitual tea consumption in contemporary MASLD populations remains uncertain.

The shape of the dose–response curve is especially important for a plant-derived beverage that combines potentially beneficial polyphenols with caffeine and other methylxanthines (15). Caffeine primarily acts through adenosine-receptor antagonism and may increase catecholaminergic activity. Its content varies by tea type and preparation, with brewed teas reported to contain approximately 14–61 mg per serving (16). Accordingly, the balance of tea constituents might differ across intake levels, supporting evaluation using non-linear rather than exclusively linear models.

Therefore, we analyzed tea consumption in the UK Biobank, focusing on MASLD. SLD was used as the overarching term encompassing its etiological subcategories. The total cohort provided an overall population benchmark, the no-SLD cohort allowed assessment of whether the observed pattern was also present in the absence of SLD, and MASLD with increased alcohol intake (MetALD) was examined separately to explore the pattern among individuals with metabolic dysfunction and higher alcohol exposure. The primary aim was to define a clinically interpretable range of tea intake in MASLD.

## 2 Materials and methods

### 2.1 Study design and participants

We conducted a prospective cohort analysis using UK Biobank baseline assessment data collected between 2006 and 2010. The UK Biobank recruited approximately 500,000 adults aged 40–69 years from assessment centers across the United Kingdom and linked baseline questionnaire, physical examination, and laboratory data to national death and hospital admission records (17). The present analysis used baseline tea consumption and longitudinal follow-up for mortality and incident clinical outcomes. All participants provided written informed consent at enrollment. This study was conducted using de-identified UK Biobank data under Application ID 117214 and was covered by approval from the North West Multi-Centre Research Ethics Committee.

Participants were sequentially excluded for missing baseline tea consumption; autoimmune hepatitis (International Classification of Diseases, 10th Revision, ICD-10 K75.4); primary biliary cholangitis (K74.3); chronic hepatitis B (B18.0–B18.1); chronic hepatitis C (B18.2); cryptogenic SLD and other predefined alternative or secondary causes of chronic liver disease/steatosis; malignancy at baseline; alcohol-associated liver disease (ICD-10 K70) or alcohol consumption exceeding 50 g/day in women or 60 g/day in men; and a history of cardiovascular disease, cerebrovascular disease, hepatic decompensation, or hepatocellular carcinoma. The final analytic cohort comprised 360,548 participants. The total cohort included all eligible participants, who were subsequently classified into three mutually exclusive subcohorts: no SLD (*n* = 146,559), MASLD (*n* = 169,241), and MetALD (*n* = 44,748).

Hepatic steatosis was assessed using the FLI, a validated population-scale marker derived from body mass index (BMI), waist circumference, triglycerides, and gamma-glutamyl transferase (GGT) (18). An FLI ≥ 60 was used to define hepatic steatosis. MASLD was defined according to the 2023 multi-society consensus nomenclature as hepatic steatosis in the presence of at least one cardiometabolic risk factor, including overweight/obesity, type 2 diabetes, hypertension, or dyslipidemia. MetALD was defined as MASLD with increased alcohol intake, corresponding to weekly alcohol consumption of 140–350 g in women and 210–420 g in men. Alcohol consumption was calculated from self-reported habitual alcohol intake at the baseline assessment (1).

### 2.2 Exposure and outcomes

Daily tea consumption was self-reported at baseline using UK Biobank field 1,488, which records the total number of cups of tea consumed per day, including decaffeinated tea. Tea subtype, brewing method, tea strength, serving size, and the addition of milk, sugar, or sweeteners were not available.

Incident disease endpoints were identified from linked hospital and registry records using predefined ICD-10 code groupings, including I20–I25 for cardiovascular disease, I60–I69 for cerebrovascular disease, and N18 for chronic kidney disease. Follow-up was measured from the baseline assessment until the first relevant event, death, or administrative censoring on 2 March 2026. Participants with the corresponding prevalent condition at baseline were excluded from the relevant incident-outcome analysis.

### 2.3 Covariates and adjustment models

Continuous variables are presented as mean ± standard deviation and categorical variables as n (%). Baseline characteristics across tea categories were compared using Welch’s one-way analysis of variance, which does not assume equal variances, for continuous variables and Pearson’s chi-squared test for categorical variables. Cox proportional hazards models estimated hazard ratios (HRs) and 95% confidence intervals (CIs). Four nested models were specified. Model 1 was crude. Model 2 adjusted for age and sex. Model 3 adjusted for BMI, diabetes, hypertension, dyslipidemia, and smoking history. Model 4 further adjusted for baseline biochemistry: aspartate aminotransferase (AST), alanine aminotransferase (ALT), GGT, platelet count, albumin, and creatinine. Covariates were selected *a priori* based on their clinical significance and plausible associations with tea consumption and the study outcomes.

### 2.4 Non-linear and sensitivity analyses

Non-linear dose–response relationships were evaluated using RCS models, with curves anchored at zero cups/day. The nadir of the J-shaped curve was estimated using complementary approaches including spline-based estimation, bootstrap resampling, and quadratic Cox models. Tea intake was modeled continuously in the RCS and quadratic Cox analyses. For bootstrap estimation, 200 resamples were generated within each cohort–outcome complete-case dataset; the spline model was refitted in each resample, and the 2.5th and 97.5th percentiles of the nadir distribution were used to derive the bootstrap 95% CIs.

To address measured confounding, IPTW was applied using baseline demographic, metabolic, smoking history, and laboratory variables. The multinomial propensity model included age, sex, BMI, diabetes, hypertension, dyslipidemia, current smoking, AST, ALT, GGT, platelet count, albumin, and log-transformed creatinine. Stabilized weights were trimmed at the 1st and 99th percentiles, robust standard errors were used, and SMD < 0.10 was considered indicative of adequate balance. Weighted Cox models were then fitted, and covariate balance was assessed using standardized mean differences (SMDs).

Predefined subgroup analyses were performed according to sex, age, BMI, diabetes, hypertension, and dyslipidemia. Additional analyses included quadratic Cox models, Poisson incidence-rate models, Weibull accelerated failure time models, and a distance-from-nadir Cox model that quantified the absolute difference between observed tea intake and the spline-derived nadir. This exploratory model was used only as a summary measure of departure from the nadir and did not distinguish between lower and higher intake relative to the nadir. Sensitivity analyses included competing-risk models for non-fatal outcomes, a 2-year landmark analysis excluding early events, and an assessment of the proportional hazards assumption. Fine–Gray subdistribution hazard models were used for non-fatal outcomes, with all-cause death treated as a competing event. The proportional hazards assumption for tea consumption was assessed using Schoenfeld residuals across cohorts and outcomes. All analyses were performed using R software version 4.5.0 (R Foundation for Statistical Computing, Vienna, Austria), and a two-sided *p-value of* < 0.05 was considered statistically significant.

## 3 Results

### 3.1 Cohort and baseline characteristics

The final cohort included 360,548 participants, of whom 169,241 met the criteria for MASLD. In the MASLD cohort, the mean age was 56.2 ± 8.0 years, 57.9% were male, and the mean BMI was 29.6 ± 4.3 kg/m^2^. Diabetes, hypertension, and dyslipidemia were present in 13.1, 47.3, and 31.5% of participants, respectively, representing the metabolic risk profile required for MASLD classification. Tea consumption was common: the 1–2, 3–4, and 5–6 cups/day categories included 37,768, 49,196, and 34,940 MASLD participants, respectively, while 17,255 participants reported ≥ 7 cups/day.

Participants with higher tea consumption were more likely to be male, whereas overall metabolic and laboratory profiles were similar across categories. Participants consuming ≥ 7 cups/day were slightly more often male (60.6%) and current smokers (15.1%) than non-consumers (55.7% male participants; 12.2% current smokers). The non-consumer group had a higher mean BMI (30.3 kg/m^2^) and slightly higher diabetes prevalence (14.5%) than the high-tea group (29.5 kg/m^2^ and 12.8%). Although differences in liver enzymes, creatinine, albumin, and platelet count across tea consumption categories were statistically significant for most comparisons, their absolute magnitudes were modest (Table 1). Similar category-wise trends were observed in the total, no-SLD, and MetALD cohorts, with modest differences in sex distribution, smoking history, and metabolic profiles across increasing tea consumption categories (Supplementary Tables 1–3). Although several baseline characteristics differed statistically across tea consumption categories, the absolute differences in most metabolic and laboratory variables were modest in this large cohort. Age, waist circumference, glucose, triglycerides, and high-density lipoprotein cholesterol (HDL-C) also showed modest differences across tea consumption categories, whereas the prevalence of hypertension and dyslipidemia varied little (Table 1). Outcome-specific event counts and analytic denominators are presented in Table 2 for the MASLD cohort and in Supplementary Tables 4–6 for the comparator cohorts.

**Table 1:** Baseline characteristics of the MASLD cohort by tea consumption category.

Characteristic	Overall N = 169,241^1^	None N = 30,082^1^	1–2 cups N = 37,768^1^	3–4 cups N = 49,196^1^	5–6 cups N = 34,940^1^	≥ 7 cups N = 17,255^1^	*p*-value^2^
Age, years	56.2 ± 8.0	54.9 ± 8.1	55.5 ± 8.2	56.9 ± 7.9	56.9 ± 7.8	56.5 ± 7.7	< 0.001
Male	98,036 (57.9%)	16,768 (55.7%)	22,444 (59.4%)	27,978 (56.9%)	20,388 (58.4%)	10,458 (60.6%)	< 0.001
BMI (kg/m^2^)	29.6 ± 4.3	30.3 ± 4.7	29.5 ± 4.3	29.3 ± 4.2	29.4 ± 4.2	29.5 ± 4.2	< 0.001
Waist circumference (cm)	97.0 ± 10.6	98.2 ± 11.1	96.9 ± 10.4	96.4 ± 10.2	96.7 ± 10.4	97.3 ± 10.7	< 0.001
Smoking history							< 0.001
Never	95,094 (56.2%)	16,488 (54.8%)	21,916 (58.0%)	28,689 (58.3%)	19,315 (55.3%)	8,686 (50.3%)	
Previous	57,474 (34.0%)	9,920 (33.0%)	12,470 (33.0%)	16,731 (34.0%)	12,391 (35.5%)	5,962 (34.6%)	
Current	16,673 (9.9%)	3,674 (12.2%)	3,382 (9.0%)	3,776 (7.7%)	3,234 (9.3%)	2,607 (15.1%)	
Diabetes	22,146 (13.1%)	4,372 (14.5%)	5,065 (13.4%)	6,242 (12.7%)	4,264 (12.2%)	2,203 (12.8%)	< 0.001
Hypertension	79,986 (47.3%)	13,880 (46.1%)	17,522 (46.4%)	23,449 (47.7%)	16,811 (48.1%)	8,324 (48.2%)	< 0.001
Dyslipidemia	53,388 (31.5%)	9,293 (30.9%)	11,711 (31.0%)	15,754 (32.0%)	11,199 (32.1%)	5,431 (31.5%)	< 0.001
Glucose (mg/dL)	93.3 ± 23.8	94.3 ± 26.5	93.4 ± 24.1	93.1 ± 22.8	92.8 ± 22.7	92.5 ± 22.7	< 0.001
Triglyceride (mg/dL)	186.0 ± 96.1	189.2 ± 99.7	187.0 ± 98.3	185.4 ± 94.2	184.3 ± 93.1	182.8 ± 95.8	< 0.001
HDL-C (mg/dL)	50.6 ± 11.9	49.9 ± 11.8	50.4 ± 11.9	51.0 ± 11.9	50.8 ± 11.9	50.5 ± 12.0	< 0.001
Creatinine (mg/dL)	0.8 ± 0.2	0.8 ± 0.2	0.8 ± 0.2	0.8 ± 0.2	0.9 ± 0.2	0.9 ± 0.2	< 0.001
AST (U/L)	27.0 ± 10.2	26.7 ± 9.3	27.2 ± 11.8	27.0 ± 9.8	27.0 ± 10.3	26.7 ± 9.1	< 0.001
ALT (U/L)	26.7 ± 15.0	27.1 ± 15.3	27.1 ± 15.7	26.5 ± 14.6	26.6 ± 14.7	26.4 ± 14.8	< 0.001
GGT (U/L)	42.1 ± 39.5	41.7 ± 38.8	42.5 ± 41.3	42.0 ± 39.1	42.1 ± 38.8	42.6 ± 39.6	0.047
Albumin, (g/dL)	4.5 ± 0.3	4.5 ± 0.3	4.5 ± 0.3	4.5 ± 0.3	4.5 ± 0.3	4.5 ± 0.3	< 0.001
Platelet (10^9^/L)	254.4 ± 59.9	256.9 ± 60.9	252.7 ± 59.2	253.9 ± 59.7	254.4 ± 60.1	255.0 ± 59.6	< 0.001
Total bilirubin (mg/dL)	0.5 ± 0.3	0.5 ± 0.2	0.5 ± 0.3	0.5 ± 0.3	0.5 ± 0.3	0.5 ± 0.2	< 0.001
Tea intake (cups/day)	3.4 ± 2.9	0.0 ± 0.0	1.6 ± 0.5	3.5 ± 0.5	5.5 ± 0.5	9.3 ± 3.5	-

^1^Mean ± SD; *n* (%).

^2^One-way analysis of means (not assuming equal variances); Pearson’s chi-squared test.

Values are mean ± SD for continuous variables and n (%) for categorical variables. *p*-values are from one-way ANOVA for continuous variables and the chi-squared test for categorical variables across tea consumption categories. BMI, body mass index; AST, aspartate aminotransferase; ALT, alanine aminotransferase; GGT, gamma-glutamyl transferase; HDL-C, high-density lipoprotein cholesterol.

**Table 2:** Cox proportional hazards models for tea consumption categories and clinical outcomes in the MASLD cohort.

Outcome	N events/N	Form	Model 1	Model 2	Model 3	Model 4
Death	16,611/169,241	None (ref)	1.00 (ref)	1.00 (ref)	1.00 (ref)	1.00 (ref)
		1–2 vs. none	0.88 (0.83–0.92) *p* < 0.001	0.80 (0.76–0.84) *p* < 0.001	0.85 (0.81–0.90) *p* < 0.001	0.85 (0.81–0.90) *p* < 0.001
		3–4 vs. none	0.98 (0.94–1.03) *p* = 0.451	0.81 (0.77–0.85) *p* < 0.001	0.88 (0.84–0.92) *p* < 0.001	0.87 (0.83–0.92) *p* < 0.001
		5–6 vs. none	0.99 (0.95–1.04) *p* = 0.770	0.82 (0.78–0.86) *p* < 0.001	0.88 (0.84–0.92) *p* < 0.001	0.86 (0.82–0.91) *p* < 0.001
		≥ 7 vs. none	1.20 (1.13–1.27) *p* < 0.001	1.02 (0.97–1.08) *p* = 0.437	1.03 (0.97–1.09) *p* = 0.291	1.00 (0.94–1.06) *p* = 0.963
Cardiovascular disease	18,004/169,238	None (ref)	1.00 (ref)	1.00 (ref)	1.00 (ref)	1.00 (ref)
		1–2 vs. none	0.95 (0.90–0.99) *P* = 0.026	0.88 (0.84–0.93) *p* < 0.001	0.93 (0.88–0.97) *p* = 0.002	0.94 (0.89–0.99) *p* = 0.016
		3–4 vs. none	1.03 (0.99–1.08) *p* = 0.165	0.90 (0.87–0.95) *p* < 0.001	0.97 (0.92–1.01) *p* = 0.139	0.97 (0.93–1.02) *p* = 0.278
		5–6 vs. none	1.05 (1.01–1.10) *p* = 0.030	0.92 (0.88–0.97) *p* < 0.001	0.97 (0.93–1.02) *p* = 0.238	0.98 (0.93–1.03) *p* = 0.345
		≥ 7 vs. none	1.18 (1.11–1.25) *p* < 0.001	1.04 (0.98–1.10) *p* = 0.159	1.08 (1.02–1.14) *p* = 0.007	1.09 (1.02–1.15) *p* = 0.006
Cerebrovascular disease	7,106/169,241	None (ref)	1.00 (ref)	1.00 (ref)	1.00 (ref)	1.00 (ref)
		1–2 vs. none	0.88 (0.82–0.95) *p* = 0.001	0.82 (0.76–0.88) *p* < 0.001	0.85 (0.79–0.91) *p* < 0.001	0.83 (0.77–0.90) *p* < 0.001
		3–4 vs. none	1.01 (0.94–1.08) *p* = 0.746	0.84 (0.78–0.90) *p* < 0.001	0.89 (0.83–0.95) *p* = 0.001	0.88 (0.81–0.94) *p* < 0.001
		5–6 vs. none	0.98 (0.91–1.05) *p* = 0.550	0.82 (0.76–0.88) *p* < 0.001	0.86 (0.79–0.92) *p* < 0.001	0.83 (0.77–0.90) *p* < 0.001
		≥ 7 vs. none	1.16 (1.06–1.26) *p* = 0.001	1.00 (0.92–1.09) *p* = 0.954	1.02 (0.93–1.11) *p* = 0.663	0.99 (0.91–1.09) *p* = 0.877
Chronic kidney disease	9,132/167,365	None (ref)	1.00 (ref)	1.00 (ref)	1.00 (ref)	1.00 (ref)
		1–2 vs. none	0.96 (0.90–1.03) *p* = 0.232	0.90 (0.84–0.96) *p* = 0.002	0.96 (0.89–1.02) *P* = 0.198	0.92 (0.86–0.99) *p* = 0.028
		3–4 vs. none	1.12 (1.05–1.19) *p* < 0.001	0.93 (0.87–0.99) *p* = 0.026	1.02 (0.96–1.08) *p* = 0.582	0.97 (0.90–1.03) *p* = 0.313
		5–6 vs. none	1.15 (1.08–1.23) *p* < 0.001	0.97 (0.91–1.04) *p* = 0.444	1.06 (0.99–1.13) *p* = 0.093	0.94 (0.87–1.01) *p* = 0.083
		≥ 7 vs. none	1.16 (1.07–1.26) *p* < 0.001	1.02 (0.94–1.11) *p* = 0.584	1.08 (1.00–1.17) *p* = 0.049	1.06 (0.97–1.15) *p* = 0.196

Hazard ratios (HR) with 95% confidence intervals (CI) from Cox proportional hazards models comparing tea consumption categories (None = reference). Model 1: crude. Model 2: adjusted for age and sex. Model 3: Model 2 + BMI, diabetes, hypertension, dyslipidemia, and smoking history. Model 4: Model 3 + AST, ALT, GGT, platelet count, albumin, and creatinine. BMI, body mass index; AST, aspartate aminotransferase; ALT, alanine aminotransferase; GGT, gamma-glutamyl transferase.

### 3.2 Categorical associations in the MASLD cohort

In the MASLD cohort, fully adjusted Cox models showed lower risks across the moderate tea consumption categories, whereas the ≥ 7 cups/day category showed attenuation of the protective association (Table 2). For all-cause mortality, in Model 4, HRs compared with no tea consumption were 0.85 (95% CI: 0.81–0.90; *p* < 0.001) for 1–2 cups/day, 0.87 (95% CI: 0.83–0.92; *p* < 0.001) for 3–4 cups/day, 0.86 (95% CI: 0.82–0.91; *p* < 0.001) for 5–6 cups/day, and 1.00 (95% CI: 0.94–1.06; *p* = 0.963) for ≥ 7 cups/day. For cardiovascular disease, 1–2 cups/day was associated with a lower estimated risk, whereas the intermediate categories did not show a consistent graded pattern. In contrast, the ≥ 7 cups/day category was associated with a modestly higher estimated cardiovascular risk than non-consumption in the fully adjusted model (HR: 1.09, 95% CI: 1.02–1.15; *p* = 0.006). Cerebrovascular disease showed lower risks across the moderate intake categories, with HRs of 0.83 (95% CI: 0.77–0.90; *p* < 0.001), 0.88 (95% CI: 0.81–0.94; *p* < 0.001), 0.83 (95% CI: 0.77–0.90; *p* < 0.001), and 0.99 (95% CI: 0.91–1.09; *p* = 0.877), respectively. Chronic kidney disease showed weaker but directionally similar estimates, with 1–2 cups/day remaining statistically significant (HR: 0.92, 95% CI: 0.86–0.99; *p* = 0.028), whereas the ≥ 7 cups/day category showed a numerically higher but non-significant HR (HR: 1.06, 95% CI: 0.97–1.15; *p* = 0.196) (Table 2). Similar overall category-wise patterns were observed in the total and no-SLD cohorts, although attenuation at higher intake levels appeared less evident than in the MASLD cohort. The MetALD cohort showed a comparatively weaker high-intake pattern overall (Supplementary Tables 4–6). Overall, the pattern was most pronounced in the MASLD cohort, where the lowest estimated risks were observed at approximately 2 cups/day, whereas attenuation of the association at higher intake levels appeared less evident in the other cohorts.

### 3.3 Dose–response and J-curve inflection

The RCS analyses showed similar dose–response patterns across the total, no-SLD, MASLD, and MetALD cohorts. In each cohort, the log hazard curve declined from zero tea intake toward the low-to-moderate range and increased thereafter. Linear per-cup Cox estimates were generally close to the null across outcomes (Figure 1). In the MASLD cohort, RCS-derived nadirs were 2.5 cups/day for all-cause mortality, 2.1 cups/day for cardiovascular disease, 2.2 cups/day for cerebrovascular disease, and 1.6 cups/day for chronic kidney disease (Figure 2). These estimates were derived from continuous RCS models of tea intake. Similar nadir ranges were observed in the total and no-SLD cohorts, whereas the MetALD cohort showed a slightly higher nadir estimate (Supplementary Figures 2–4). Across cohorts and outcomes, nadir estimates were generally clustered approximately 2 cups/day, with corresponding category-wise crude HR patterns across tea intake levels (Supplementary Figure 5). Overall, the dose–response curves consistently suggested the lowest estimated risk within the range of approximately 2 cups/day across cohorts and outcomes.

**Figure 1:**
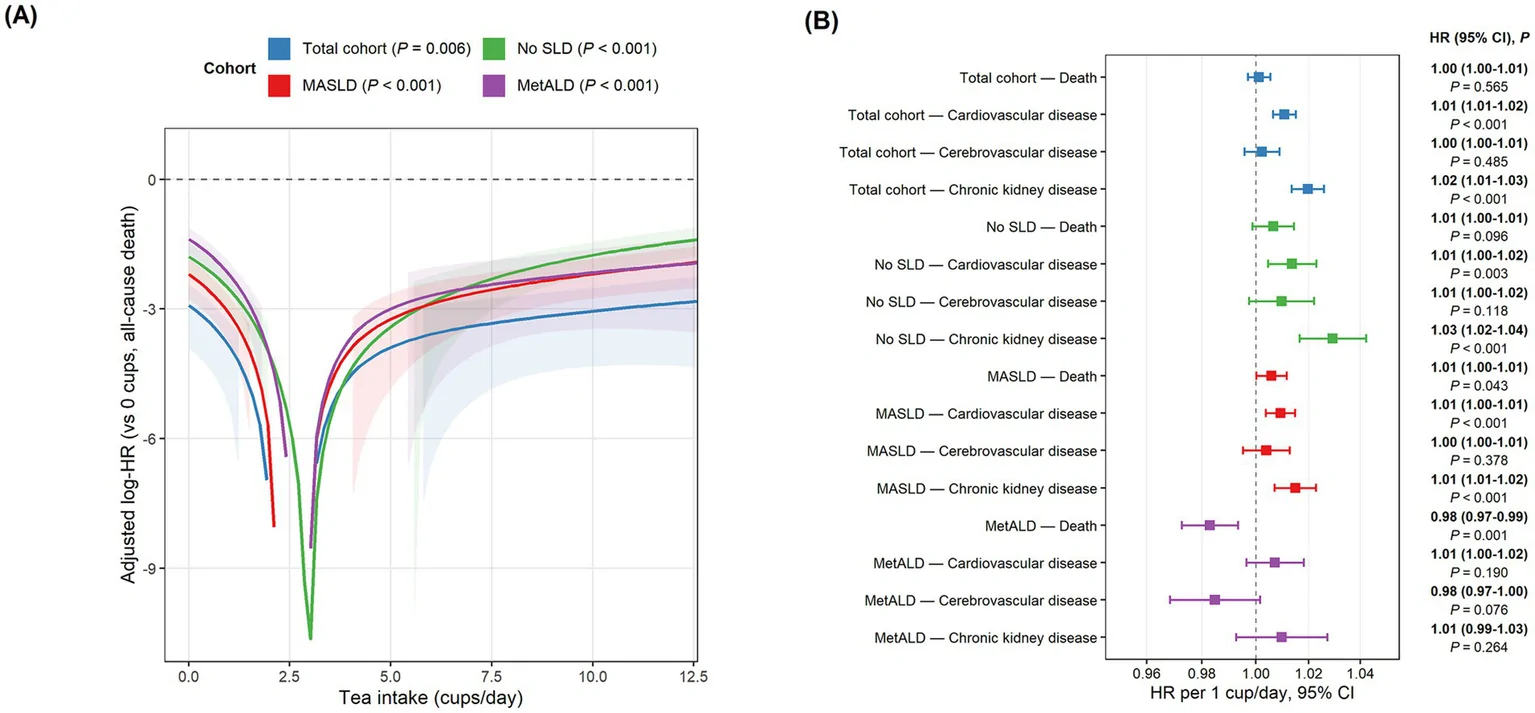
Cross-cohort dose–response analyses of tea consumption. **(A)** RCS curves for all-cause mortality across the four cohorts. **(B)** Adjusted HRs per 1 cup/day of tea across four cohorts and four clinical outcomes. RCS, restricted cubic spline; HR, hazard ratio; CI, confidence interval; SLD, steatotic liver disease; MASLD, metabolic dysfunction-associated steatotic liver disease; MetALD, MASLD with increased alcohol intake.

**Figure 2:**
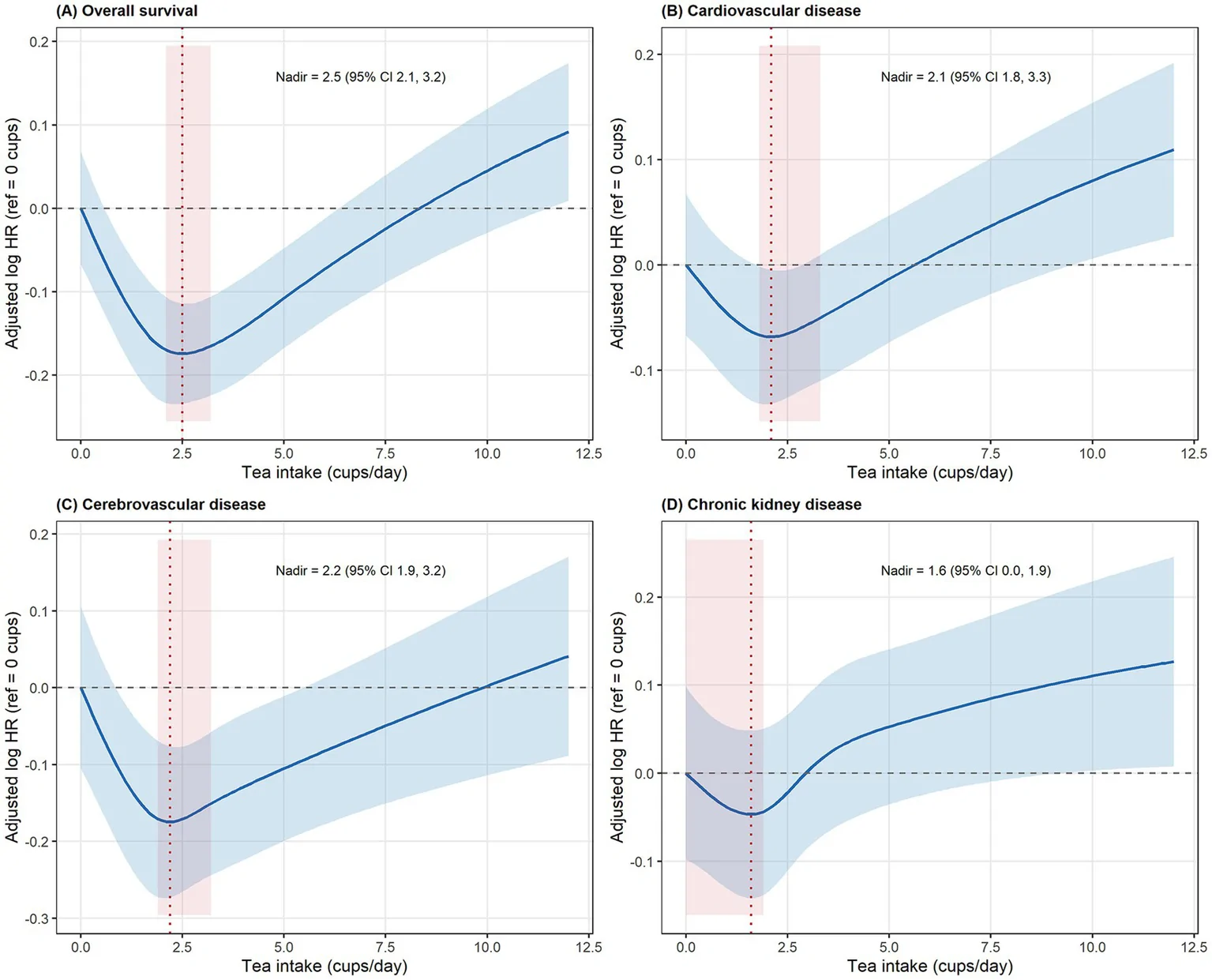
Dose–response curves of tea consumption in the MASLD cohort. RCS-derived dose–response curves for **(A)** all-cause mortality, **(B)** cardiovascular disease, **(C)** cerebrovascular disease, and **(D)** chronic kidney disease in MASLD. Red vertical lines indicate the estimated nadirs. RCS, restricted cubic spline; HR, hazard ratio; MASLD, metabolic dysfunction-associated steatotic liver disease.

### 3.4 Survival curves and subgroup analyses

Kaplan–Meier and cumulative incidence curves across the five tea consumption categories showed separation between intake groups during follow-up (Figure 3). In the MASLD cohort, the 1–2, 3–4, and 5–6 cups/day groups generally showed lower event rates than the no-tea group, whereas the ≥7 cups/day group showed less separation from the reference category across outcomes. Global log-rank tests comparing all five tea consumption categories were significant for all four outcomes (all *p* < 0.001) (Figure 3).

**Figure 3:**
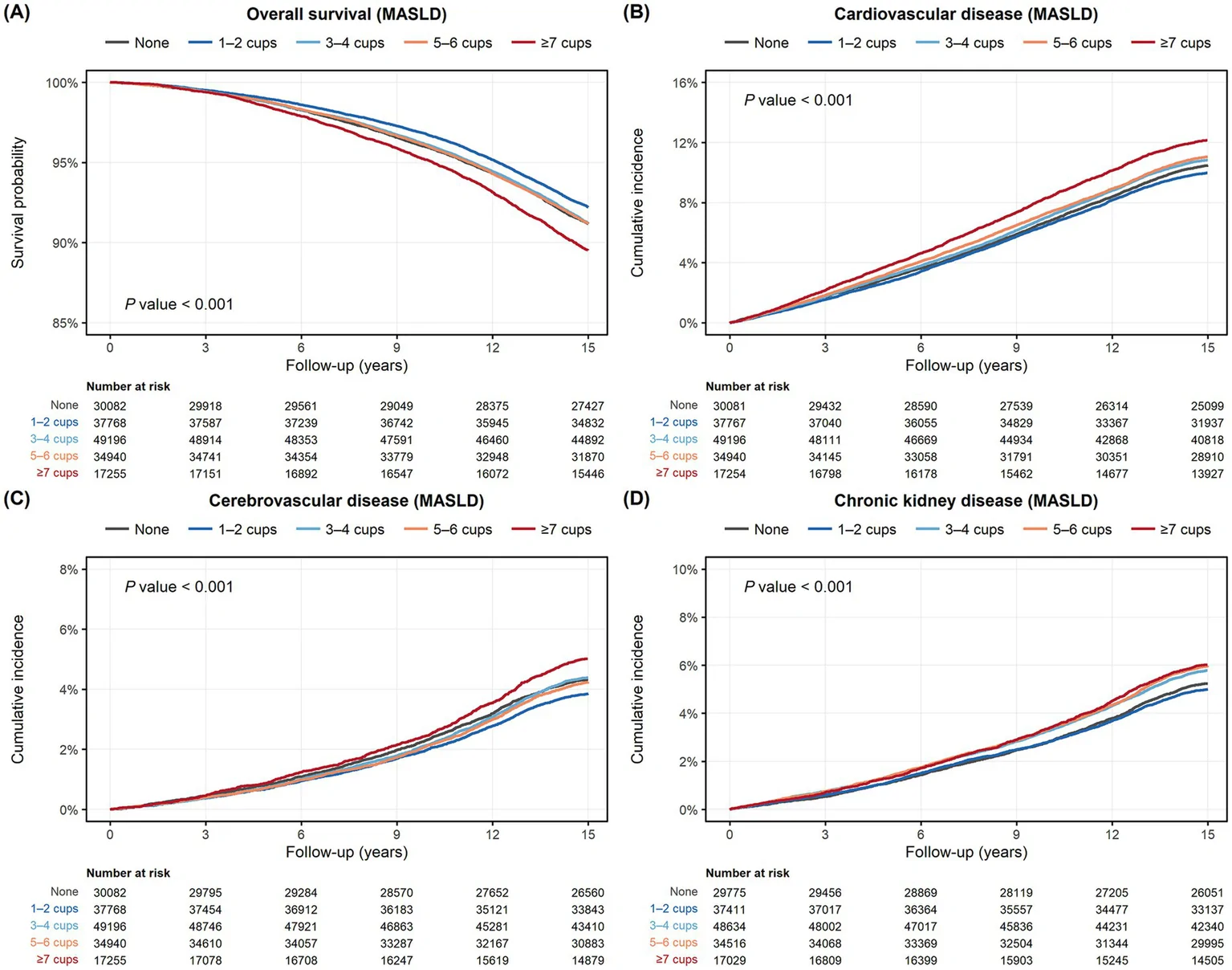
Kaplan–Meier survival and cumulative incidence curves by tea consumption category in MASLD. **(A)** Overall survival, **(B)** cardiovascular disease, **(C)** cerebrovascular disease, and **(D)** chronic kidney disease according to five tea consumption categories. MASLD, metabolic dysfunction-associated steatotic liver disease.

Exploratory subgroup analyses showed broadly similar directions of association across sex, age, BMI, diabetes, hypertension, and dyslipidemia strata (Figure 4). However, formal interaction testing was not performed, and apparent differences between subgroup-specific estimates should be interpreted cautiously. Category-wise separation was also observed in the total and no-SLD cohorts, whereas the MetALD cohort showed less distinct separation across intake groups (Supplementary Figures 6–8). Corresponding exploratory subgroup analyses in the total, no SLD, and MetALD cohorts are shown (Supplementary Figures 9–11).

**Figure 4:**
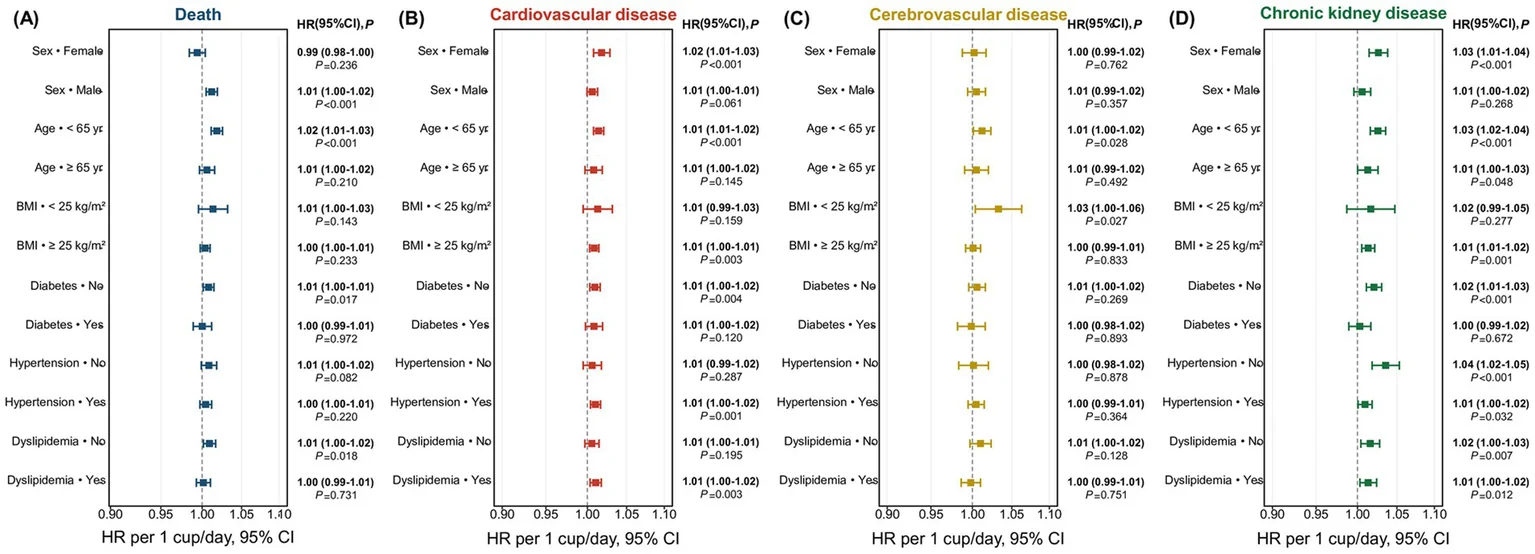
Subgroup analyses of tea consumption in the MASLD cohort. Forest plots showing adjusted hazard ratios per 1 cup/day of tea across predefined subgroups for **(A)** all-cause mortality, **(B)** cardiovascular disease, **(C)** cerebrovascular disease, and **(D)** chronic kidney disease. HR, hazard ratio; CI, confidence interval; BMI, body mass index; MASLD, metabolic dysfunction-associated steatotic liver disease.

### 3.5 Post-IPTW analyses

To reduce baseline imbalance across tea consumption categories, IPTW analyses were performed. IPTW reduced baseline imbalance across tea consumption categories in all cohorts. In the MASLD cohort, post-weighting SMDs were ≤ 0.022, below the prespecified threshold of 0.10 for adequate balance, with similarly low residual imbalance observed in the other cohorts (Supplementary Tables 7–10; Supplementary Figure 12). IPTW-weighted Cox analyses showed patterns generally similar to those in the primary analyses. In the MASLD cohort, HRs for all-cause mortality compared with no tea consumption were 0.83 (95% CI: 0.76–0.90; *p* < 0.001), 0.81 (95% CI: 0.75–0.88; *p* < 0.001), 0.89 (95% CI: 0.82–0.96; *p* = 0.005), and 0.94 (95% CI: 0.85–1.04; *p* = 0.210) for 1–2, 3–4, 5–6, and ≥ 7 cups/day, respectively. For cardiovascular disease, the IPTW estimate for the ≥ 7 cups/day category was 1.06 (95% CI: 0.96–1.16; *p* = 0.231), indicating that the higher cardiovascular estimate observed in the primary analysis was attenuated and was not statistically significant after weighting. IPTW-weighted spline analyses continued to show nadir estimates near 2 cups/day across cohorts and outcomes (Figure 5; Supplementary Table 11). Similar overall IPTW-weighted patterns were observed in the total and no-SLD cohorts, whereas the MetALD cohort showed less distinct attenuation at higher intake levels (Supplementary Figure 13; Supplementary Table 11). IPTW-weighted Kaplan–Meier and cumulative incidence curves also showed persistent separation between tea intake categories during follow-up, particularly in the MASLD cohort (Supplementary Figure 14). Overall, IPTW analyses supported a lower-risk pattern at low-to-moderate intake levels, while the higher cardiovascular estimate at ≥7 cups/day in the primary model was not statistically significant after weighting.

**Figure 5:**
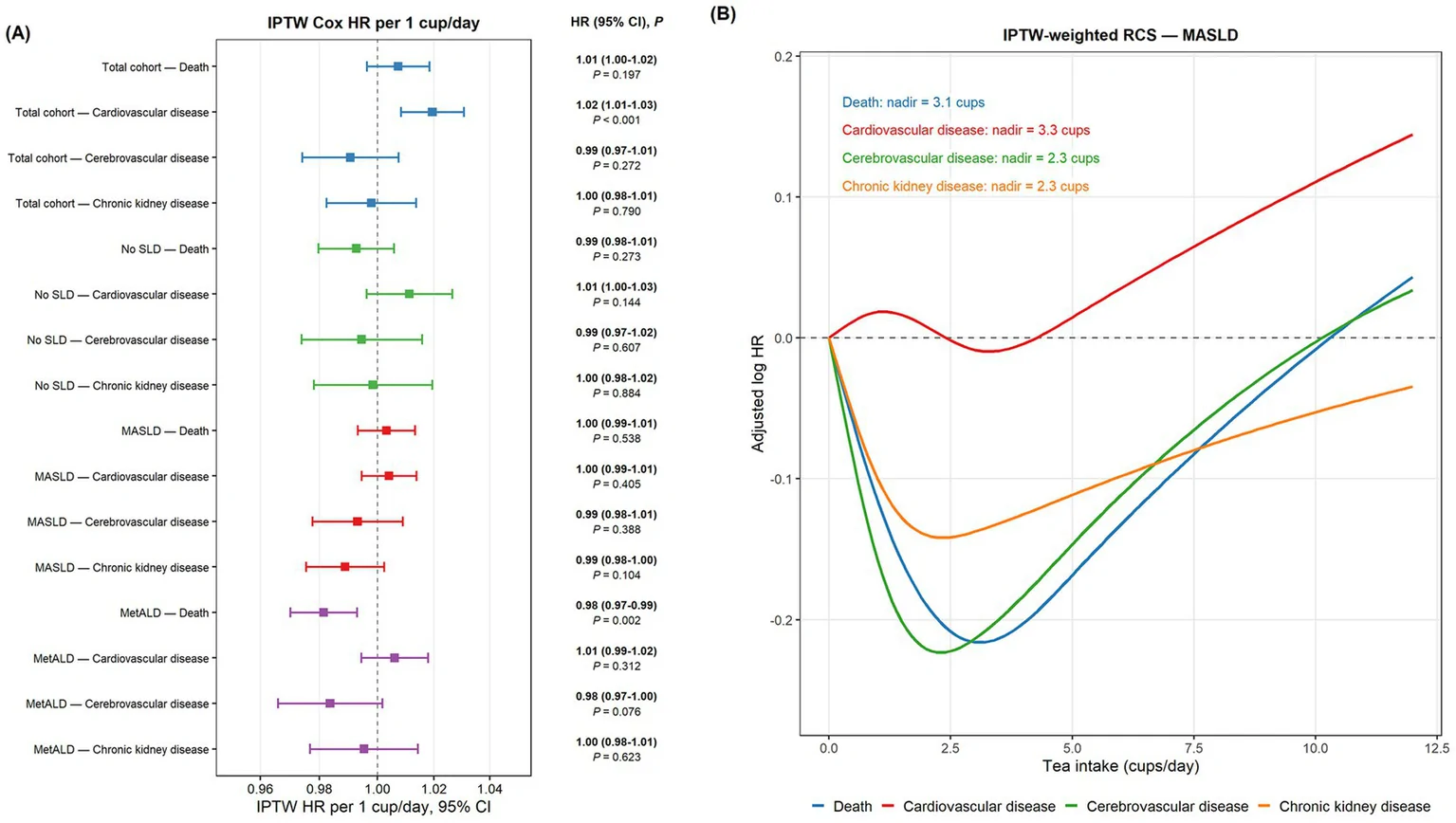
IPTW analyses of tea consumption. **(A)** IPTW-weighted hazard ratios per 1 cup/day of tea across four cohorts and four clinical outcomes. **(B)** IPTW-weighted RCS dose–response curves across four clinical outcomes in the MASLD cohort. RCS, restricted cubic spline; IPTW, inverse probability of treatment weighting; HR, hazard ratio; CI, confidence interval; MASLD, metabolic dysfunction-associated steatotic liver disease.

### 3.6 Multi-method and sensitivity analyses

Additional modeling approaches showed patterns generally similar to those in the primary analyses. Quadratic Cox models supported non-linear associations for mortality and vascular outcomes across cohorts, whereas chronic kidney disease showed less pronounced curvature (Figure 6A; Supplementary Figures 15, 16). In the distance-from-nadir Cox model, using the RCS-derived optimum of approximately 2 cups/day as the nadir, each 1-cup/day increase in the absolute deviation from the nadir was associated with a higher risk of all-cause mortality (HR, 1.026; 95% CI, 1.019–1.034; *p* < 0.001), cardiovascular disease (HR, 1.019; 95% CI, 1.012–1.027; *p* < 0.001), cerebrovascular disease (HR, 1.023; 95% CI, 1.011–1.034; *p* < 0.001), and chronic kidney disease (HR, 1.021; 95% CI, 1.011–1.031; *p* < 0.001) in the MASLD cohort (Figure 6B and Supplementary Table 12). Because this exploratory measure combines intake below and above the nadir, it should be interpreted only as a summary of absolute departure from the estimated nadir, rather than as evidence of equivalent effects on either side of the curve. Poisson incidence-rate and Weibull accelerated failure time models showed generally comparable overall patterns, although the corresponding average linear estimates remained close to the null (Figures 6C,D; Supplementary Figure 15).

**Figure 6:**
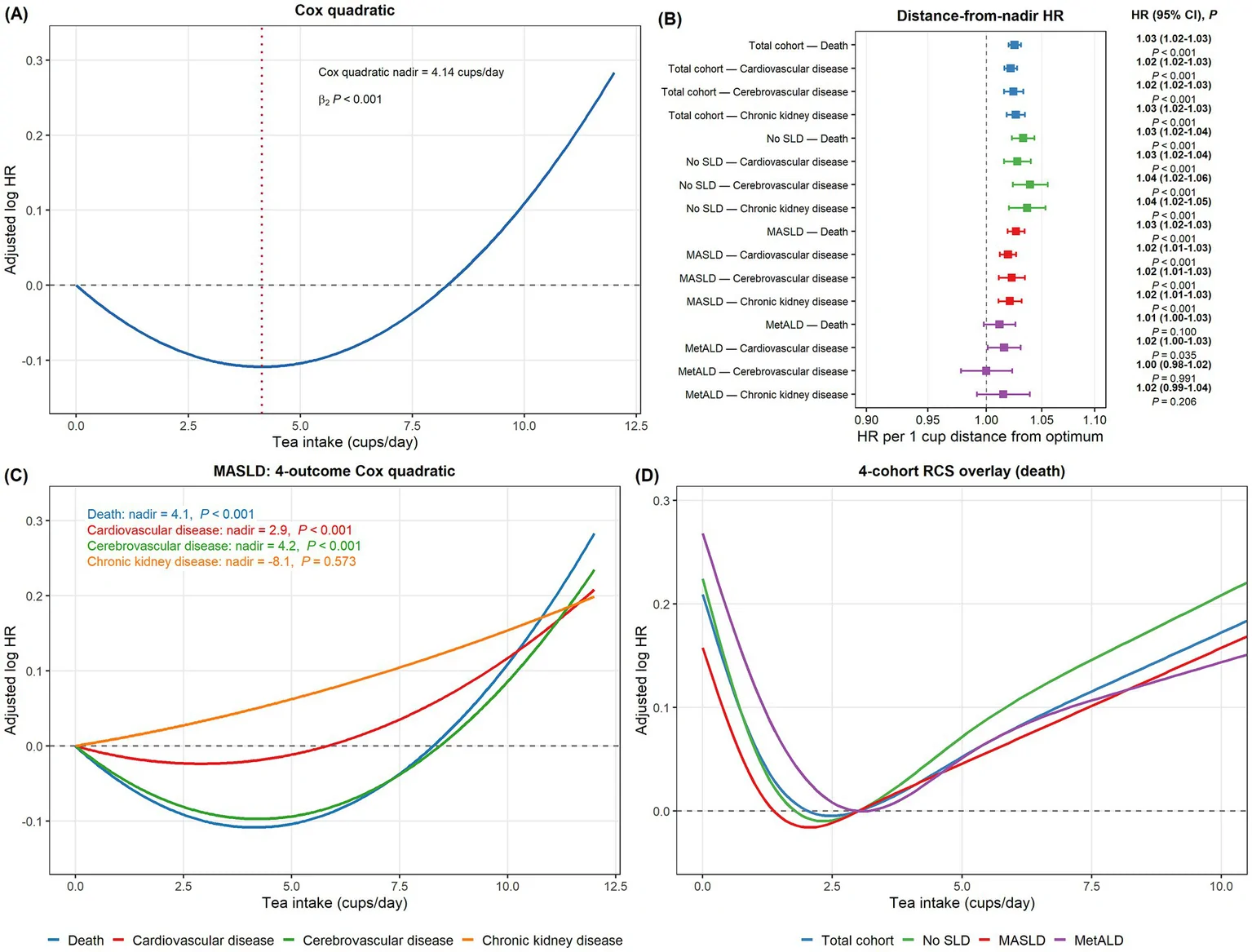
Multi-method validation of the tea dose–response relationship. **(A)** Quadratic Cox model, **(B)** distance-from-nadir Cox model, **(C)** quadratic dose–response curves across four outcomes in MASLD, and **(D)** cross-cohort RCS curves for all-cause mortality. RCS, restricted cubic spline; HR, hazard ratio; CI, confidence interval; MASLD, metabolic dysfunction-associated steatotic liver disease; MetALD, MASLD with increased alcohol intake.

Fine–Gray subdistribution hazard analyses for non-fatal outcomes showed similar category-wise directions after treating all-cause death as a competing event (Supplementary Figure 16). The 2-year landmark analysis, which was selected to reduce the potential influence of early events and reverse causation while retaining sufficient follow-up, also yielded estimates generally comparable to those of the primary analyses after exclusion of early events (Supplementary Figure 17). Schoenfeld residual testing showed no significant violation of the proportional hazards assumption for tea consumption categories across cohorts and outcomes; all exposure-specific *p*-values exceeded 0.05 (Supplementary Figure 18). Overall, these additional analyses showed findings generally consistent with the primary dose–response analyses across cohorts and outcomes.

## 4 Discussion

The present large cohort analysis of the UK Biobank showed non-linear associations between tea consumption and all-cause mortality, cardiovascular disease, cerebrovascular disease, and chronic kidney disease in MASLD. Across multiple analytical approaches, the lowest estimated risks were observed within a low-to-moderate intake range, although the estimated nadir varied across outcomes and analytical approaches. These observational findings should not be interpreted as identifying an optimal dose or establishing causality.

The biological coherence of the observed curve is supported by existing mechanistic and clinical literature. Tea-derived phytochemicals might influence several pathways important to MASLD, including oxidative stress, vascular dysfunction, inflammation, and lipid metabolism (19, 20). Experimental and clinical studies conducted during the non-alcoholic fatty liver disease era, when NAFLD was the nomenclature used for the condition now termed MASLD, have reported favorable effects of green tea or catechin-rich preparations on liver enzymes, hepatic fat accumulation, oxidative stress, and inflammatory signaling, while population-based meta-analyses have suggested inverse associations between tea consumption and cardiovascular disease or all-cause mortality (11, 12, 21, 22). However, these data do not establish that the associations observed in the present study are attributable to specific tea constituents or that higher tea intake causes either benefit or harm.

Caffeine and other tea constituents might contribute to heterogeneous associations across intake levels, but caffeine intake and tea composition were not measured in this study. Concentrated green tea extracts and high-dose epigallocatechin-3-gallate preparations have been associated with hepatotoxicity in susceptible individuals (23). However, tea composition and epigallocatechin-3-gallate exposure were not measured in this study, and evidence from concentrated supplements cannot be directly extrapolated to habitual tea consumption. Accordingly, we cannot attribute the estimates at higher tea intake to caffeine exposure, adenosine-receptor antagonism, or other specific mechanisms. Tea polyphenols may influence iron absorption, although the clinical relevance of this pathway in the present population could not be assessed. Similarly, unmeasured characteristics related to tea consumption, including dietary habits, sleep, work patterns, and beverage additives, may contribute to residual confounding; these possibilities should be regarded as limitations of exposure measurement and covariate ascertainment rather than as established explanations for the observed associations (24–27).

The cohort comparisons were descriptive and were not designed to establish MASLD-specific biological susceptibility or formal between-cohort differences in the dose–response relationship. The more apparent attenuation pattern observed in MASLD may reflect differences in baseline cardiometabolic risk, residual confounding, exposure measurement, or statistical precision rather than a MASLD-specific biological response. Similarly, the less distinct pattern in MetALD may reflect its smaller sample size, wider CIs, different baseline characteristics, and differing alcohol exposure patterns. Therefore, these findings should be interpreted as exploratory. In the primary adjusted analysis, the ≥7 cups/day category was associated with a modestly higher estimated cardiovascular risk than non-consumption. However, this estimate was attenuated and no longer statistically significant after IPTW. Thus, the apparent high-intake cardiovascular association should be interpreted cautiously.

Major strengths include the large prospective cohort, hard clinical endpoints, evaluation of non-linearity, complementary analytical approaches, and inclusion of comparator cohorts within the contemporary SLD framework. Nevertheless, several limitations should be acknowledged. Tea consumption was self-reported at baseline without information on tea subtype, brewing characteristics, or longitudinal changes. Residual confounding by alcohol consumption below the MetALD threshold, diet quality, physical activity, socioeconomic status, education, and other lifestyle characteristics remains possible despite multivariable adjustment and IPTW analyses. MASLD was identified using a validated population-scale algorithm rather than imaging or biopsy. At the conventional FLI threshold of ≥60, the original validation study reported high specificity for hepatic steatosis (approximately 86%) but moderate sensitivity (approximately 61%) (18). Therefore, some participants with steatosis may have been classified in the no-SLD cohort, which could have influenced between-cohort comparisons. In addition, UK Biobank participants might be healthier and more health-conscious than the source population, were recruited at ages 40–69 years, and have limited ethnic diversity. Therefore, the findings may not be directly generalizable to younger adults, more ethnically diverse populations, or patients with advanced liver disease. Finally, the observational design supports associations rather than definitive causal reference.

In conclusion, tea consumption showed a consistent non-linear association with mortality and major cardiometabolic outcomes in MASLD, with the lowest estimated risks within a low-to-moderate intake range. The exact nadir varied across outcomes, and the higher cardiovascular estimate observed at ≥ 7 cups/day was not statistically significant after IPTW. These observational findings do not establish an optimal intake or causal benefit.

## Data Availability

The data analyzed in this study is subject to the following licenses/restrictions: the dataset used in this study is available from the UK Biobank under Application ID 117214. Access is restricted and requires approval from the UK Biobank; therefore, the data are not publicly available. Requests to access these datasets should be directed to https://www.ukbiobank.ac.uk/.
