# Supplementary material for: Dose–response relationship between tea consumption and mortality, cardiovascular, cerebrovascular, and renal outcomes in metabolic dysfunction-associated steatotic liver disease: a prospective cohort study
Source: Front Nutr. 2026 Sep 24;13:1945413. doi: 10.3389/fnut.2026.1945413 (PMC13646909; doi:10.3389/fnut.2026.1945413)
Supplement: Supplementary file 1 [file Supplementary_file_1.docx]

Supplementary Material

# 1 Supplementary Figures and Tables

## 1.1 Supplementary Figures

**Supplementary Fig. 1. Study flowchart.** Cohort selection and exclusion cascade for the UK Biobank tea consumption analysis. The final analytic cohort was stratified into total, no SLD, MASLD, and MetALD cohorts. SLD, steatotic liver disease; MASLD, metabolic dysfunction-associated steatotic liver disease; MetALD, metabolic dysfunction-associated steatotic liver disease with increased alcohol intake.

**Supplementary Fig. 2. Dose-response curves of tea consumption in the total cohort.** RCS-derived dose-response curves for (A) all-cause mortality, (B) cardiovascular disease, (C) cerebrovascular disease, and (D) chronic kidney disease. Blue lines indicate adjusted HRs, and blue shaded areas represent 95% CIs. Red vertical lines indicate estimated nadirs, and red shaded bands represent bootstrap 95% CIs of the nadirs. RCS, restricted cubic spline; HR, hazard ratio; CI, confidence interval.

**Supplementary Fig. 3. Dose-response curves of tea consumption in the no SLD cohort.** RCS-derived dose-response curves for (A) all-cause mortality, (B) cardiovascular disease, (C) cerebrovascular disease, and (D) chronic kidney disease. Blue lines indicate adjusted HRs, and blue shaded areas represent 95% CIs. Red vertical lines indicate estimated nadirs, and red shaded bands represent bootstrap 95% CIs of the nadirs. RCS, restricted cubic spline; HR, hazard ratio; CI, confidence interval; SLD, steatotic liver disease.

**Supplementary Fig. 4. Dose-response curves of tea consumption in the MetALD cohort.** RCS-derived dose-response curves for (A) all-cause mortality, (B) cardiovascular disease, (C) cerebrovascular disease, and (D) chronic kidney disease. Blue lines indicate adjusted HRs, and blue shaded areas represent 95% CIs. Red vertical lines indicate estimated nadirs, and red shaded bands represent bootstrap 95% CIs of the nadirs. RCS, restricted cubic spline; HR, hazard ratio; CI, confidence interval; MetALD, metabolic dysfunction-associated steatotic liver disease with increased alcohol intake.

**Supplementary Fig. 5. Tea consumption distribution and category-based crude HRs across cohorts.** Histograms of tea consumption and crude HRs by tea intake category are shown for the total, no SLD, MASLD, and MetALD cohorts. HR, hazard ratio; SLD, steatotic liver disease; MASLD, metabolic dysfunction-associated steatotic liver disease; MetALD, metabolic dysfunction-associated steatotic liver disease with increased alcohol intake.

**Supplementary Fig. 6. Kaplan–Meier survival and cumulative incidence curves by tea consumption category in the total cohort.** (A) Overall survival, (B) cardiovascular disease, (C) cerebrovascular disease, and (D) chronic kidney disease according to five tea consumption categories.

**Supplementary Fig. 7. Kaplan–Meier survival and cumulative incidence curves by tea consumption category in the no SLD cohort.** (A) Overall survival, (B) cardiovascular disease, (C) cerebrovascular disease, and (D) chronic kidney disease according to five tea consumption categories. SLD, steatotic liver disease.

**Supplementary Fig. 8. Kaplan–Meier survival and cumulative incidence curves by tea consumption category in the MetALD cohort.** (A) Overall survival, (B) cardiovascular disease, (C) cerebrovascular disease, and (D) chronic kidney disease according to five tea consumption categories. MetALD, metabolic dysfunction-associated steatotic liver disease with increased alcohol intake.

**Supplementary Fig. 9. Subgroup analyses of tea consumption in the total cohort.** Forest plots showing adjusted HRs per 1 cup/day of tea across predefined subgroups for (A) all-cause mortality, (B) cardiovascular disease, (C) cerebrovascular disease, and (D) chronic kidney disease. HR, hazard ratio; CI, confidence interval; BMI, body mass index.

**Supplementary Fig. 10. Subgroup analyses of tea consumption in the no SLD cohort.** Forest plots showing adjusted HRs per 1 cup/day of tea across predefined subgroups for (A) all-cause mortality, (B) cardiovascular disease, (C) cerebrovascular disease, and (D) chronic kidney disease. HR, hazard ratio; CI, confidence interval; BMI, body mass index; SLD, steatotic liver disease.

**Supplementary Fig. 11. Subgroup analyses of tea consumption in the MetALD cohort.** Forest plots showing adjusted HRs per 1 cup/day of tea across predefined subgroups for (A) all-cause mortality, (B) cardiovascular disease, (C) cerebrovascular disease, and (D) chronic kidney disease. HR, hazard ratio; CI, confidence interval; BMI, body mass index; MetALD, metabolic dysfunction-associated steatotic liver disease with increased alcohol intake.

**Supplementary Fig. 12. Covariate balance before and after IPTW. Standardized mean differences before and after IPTW are shown for the total, no SLD, MASLD, and MetALD cohorts.** IPTW, inverse probability of treatment weighting; SMD, standardized mean difference; SLD, steatotic liver disease; MASLD, metabolic dysfunction-associated steatotic liver disease; MetALD, metabolic dysfunction-associated steatotic liver disease with increased alcohol intake.

**Supplementary Fig. 13. IPTW-weighted RCS dose-response curves in non-MASLD cohorts.** IPTW-weighted RCS curves across four clinical outcomes are shown for (A) total cohort, (B) no SLD cohort, and (C) MetALD cohort. RCS, restricted cubic spline; IPTW, inverse probability of treatment weighting; HR, hazard ratio; SLD, steatotic liver disease; MetALD, metabolic dysfunction-associated steatotic liver disease with increased alcohol intake.

**Supplementary Fig. 14. IPTW-weighted Kaplan–Meier survival curves across cohorts.** IPTW-weighted overall survival curves by tea consumption category are shown for (A) total cohort, (B) no SLD cohort, (C) MASLD cohort, and (D) MetALD cohort. IPTW, inverse probability of treatment weighting; SLD, steatotic liver disease; MASLD, metabolic dysfunction-associated steatotic liver disease; MetALD, metabolic dysfunction-associated steatotic liver disease with increased alcohol intake.

**Supplementary Fig. 15. Quadratic Cox dose-response curves in non-MASLD cohorts.** Quadratic Cox curves across four clinical outcomes are shown for (A) total cohort, (B) no SLD cohort, and (C) MetALD cohort. HR, hazard ratio; SLD, steatotic liver disease; MetALD, metabolic dysfunction-associated steatotic liver disease with increased alcohol intake.

**Supplementary Fig. 16. Fine–Gray competing-risk analyses for non-fatal outcomes.** Cumulative incidence curves for cardiovascular disease, cerebrovascular disease, and chronic kidney disease are shown across four cohorts, with all-cause death treated as a competing event. SLD, steatotic liver disease; MASLD, metabolic dysfunction-associated steatotic liver disease; MetALD, metabolic dysfunction-associated steatotic liver disease with increased alcohol intake.

**Supplementary Fig. 17. Two-year landmark sensitivity analysis.** Cox and survival analyses were repeated after excluding events occurring within the first 2 years of follow-up across cohorts and outcomes. SLD, steatotic liver disease; MASLD, metabolic dysfunction-associated steatotic liver disease; MetALD, metabolic dysfunction-associated steatotic liver disease with increased alcohol intake.

**Supplementary Fig. 18. Schoenfeld residual test for the proportional hazards assumption.** Schoenfeld residual test results are shown across four cohorts and four clinical outcomes. The dashed line indicates *P* = 0.05. PH, proportional hazards; BMI, body mass index; SLD, steatotic liver disease; MASLD, metabolic dysfunction-associated steatotic liver disease; MetALD, metabolic dysfunction-associated steatotic liver disease with increased alcohol intake.

## 1.2 Supplementary Tables

**Supplementary Table 1. Baseline characteristics of the total cohort by tea consumption category.**

| **Characteristic** | **Overall**  N = 360,548^1^ | **None**  N = 63,284^1^ | **1-2 cups**  N = 83,922^1^ | **3-4 cups**  N = 106,526^1^ | **5-6 cups**  N = 72,257^1^ | **≥ 7 cups**  N = 34,559^1^ | ***P* value**^2^ |
| --- | --- | --- | --- | --- | --- | --- | --- |
| **Age, years** | 55.8 ± 8.1 | 54.7 ± 8.2 | 55.4 ± 8.3 | 56.4 ± 8.0 | 56.3 ± 7.9 | 56.0 ± 7.8 | < 0.001 |
| **Male** | 163,023 (45.2%) | 28,002 (44.2%) | 39,203 (46.7%) | 46,889 (44.0%) | 32,499 (45.0%) | 16,430 (47.5%) | < 0.001 |
| **BMI (kg/m²)** | 27.0 ± 4.6 | 27.5 ± 5.0 | 26.9 ± 4.5 | 26.8 ± 4.4 | 26.9 ± 4.4 | 27.0 ± 4.6 | < 0.001 |
| **Waist circumference (cm)** | 89.0 ± 13.2 | 90.0 ± 13.9 | 89.0 ± 13.1 | 88.5 ± 12.9 | 88.8 ± 13.0 | 89.3 ± 13.4 | < 0.001 |
| **Smoking status** |  |  |  |  |  |  | < 0.001 |
| Never | 203,754 (56.5%) | 34,370 (54.3%) | 47,998 (57.2%) | 62,274 (58.5%) | 41,155 (57.0%) | 17,957 (52.0%) |  |
| Previous | 120,368 (33.4%) | 20,776 (32.8%) | 28,207 (33.6%) | 35,905 (33.7%) | 24,223 (33.5%) | 11,257 (32.6%) |  |
| Current | 36,426 (10.1%) | 8,138 (12.9%) | 7,717 (9.2%) | 8,347 (7.8%) | 6,879 (9.5%) | 5,345 (15.5%) |  |
| **Diabetes** | 30,615 (8.5%) | 6,003 (9.5%) | 7,289 (8.7%) | 8,607 (8.1%) | 5,759 (8.0%) | 2,957 (8.6%) | < 0.001 |
| **Hypertension** | 137,900 (38.2%) | 23,704 (37.5%) | 31,507 (37.5%) | 40,962 (38.5%) | 28,107 (38.9%) | 13,620 (39.4%) | < 0.001 |
| **Dyslipidemia** | 90,450 (25.1%) | 15,630 (24.7%) | 20,890 (24.9%) | 26,930 (25.3%) | 18,236 (25.2%) | 8,764 (25.4%) | 0.025 |
| **Glucose (mg/dL)** | 91.4 ± 20.5 | 91.9 ± 22.3 | 91.6 ± 20.7 | 91.3 ± 19.7 | 91.2 ± 19.8 | 91.0 ± 20.4 | < 0.001 |
| **Triglyceride (mg/dL)** | 150.2 ± 89.1 | 153.6 ± 93.2 | 151.0 ± 90.8 | 149.1 ± 87.1 | 148.7 ± 86.8 | 148.2 ± 88.2 | < 0.001 |
| **HDL-C (mg/dL)** | 56.9 ± 14.8 | 56.2 ± 14.9 | 57.1 ± 15.0 | 57.5 ± 14.8 | 56.9 ± 14.6 | 56.3 ± 14.6 | < 0.001 |
| **Creatinine (mg/dL)** | 0.8 ± 0.2 | 0.8 ± 0.2 | 0.8 ± 0.2 | 0.8 ± 0.2 | 0.8 ± 0.2 | 0.8 ± 0.2 | < 0.001 |
| **AST (U/L)** | 25.9 ± 9.7 | 25.8 ± 10.2 | 26.0 ± 10.8 | 25.8 ± 9.1 | 25.8 ± 9.2 | 25.7 ± 9.0 | < 0.001 |
| **ALT (U/L)** | 23.0 ± 13.6 | 23.5 ± 14.3 | 23.3 ± 14.3 | 22.8 ± 13.0 | 22.8 ± 13.1 | 22.8 ± 13.3 | < 0.001 |
| **GGT (U/L)** | 35.2 ± 36.7 | 35.8 ± 38.0 | 35.8 ± 38.7 | 34.8 ± 36.2 | 34.8 ± 34.5 | 35.2 ± 35.1 | < 0.001 |
| **Albumin, (g/dL)** | 4.5 ± 0.3 | 4.5 ± 0.3 | 4.5 ± 0.3 | 4.5 ± 0.3 | 4.5 ± 0.3 | 4.5 ± 0.3 | < 0.001 |
| **Platelet (10⁹/L)** | 253.7 ± 59.0 | 255.7 ± 59.9 | 252.2 ± 58.3 | 252.9 ± 58.5 | 254.2 ± 59.3 | 255.7 ± 60.0 | < 0.001 |
| **Total bilirubin (mg/dL)** | 0.5 ± 0.3 | 0.5 ± 0.2 | 0.5 ± 0.3 | 0.5 ± 0.3 | 0.5 ± 0.3 | 0.5 ± 0.3 | < 0.001 |
| **Tea intake (cups/day)** | 3.4 ± 2.9 | 0.0 ± 0.0 | 1.6 ± 0.5 | 3.5 ± 0.5 | 5.4 ± 0.5 | 9.2 ± 3.5 | - |
| ^1^Mean ± SD; n (%) | | | | | | | |
| ^2^One-way analysis of means (not assuming equal variances); Pearson's Chi-squared test | | | | | | | |
| Values are mean ± SD for continuous variables and n (%) for categorical variables. *P* values are from one-way ANOVA for continuous variables and chi-squared test for categorical variables across tea consumption categories. BMI, body mass index; AST, aspartate aminotransferase; ALT, alanine aminotransferase; GGT, gamma-glutamyl transferase; HDL-C, high-density lipoprotein cholesterol. | | | | | | | |

**Supplementary Table 2. Baseline characteristics of the no SLD cohort by tea consumption category.**

| **Characteristic** | **Overall**  N = 146,559^1^ | **None**  N = 24,553^1^ | **1-2 cups**  N = 34,097^1^ | **3-4 cups**  N = 44,310^1^ | **5-6 cups**  N = 29,623^1^ | **≥ 7 cups**  N = 13,976^1^ | ***P* value**^2^ |
| --- | --- | --- | --- | --- | --- | --- | --- |
| **Age, years** | 54.6 ± 8.2 | 53.5 ± 8.2 | 54.2 ± 8.4 | 55.2 ± 8.2 | 55.1 ± 8.0 | 54.9 ± 7.9 | < 0.001 |
| **Male** | 33,417 (22.8%) | 5,315 (21.6%) | 8,102 (23.8%) | 9,857 (22.2%) | 6,624 (22.4%) | 3,519 (25.2%) | < 0.001 |
| **BMI (kg/m²)** | 23.5 ± 2.3 | 23.6 ± 2.4 | 23.5 ± 2.3 | 23.5 ± 2.3 | 23.6 ± 2.3 | 23.6 ± 2.4 | < 0.001 |
| **Waist circumference (cm)** | 77.6 ± 7.1 | 77.4 ± 7.1 | 77.6 ± 7.1 | 77.5 ± 7.0 | 77.6 ± 7.1 | 77.7 ± 7.1 | 0.005 |
| **Smoking status** |  |  |  |  |  |  | < 0.001 |
| Never | 90,995 (62.1%) | 14,550 (59.3%) | 21,252 (62.3%) | 28,372 (64.0%) | 18,773 (63.4%) | 8,048 (57.6%) |  |
| Previous | 41,930 (28.6%) | 6,925 (28.2%) | 10,074 (29.5%) | 12,925 (29.2%) | 8,241 (27.8%) | 3,765 (26.9%) |  |
| Current | 13,634 (9.3%) | 3,078 (12.5%) | 2,771 (8.1%) | 3,013 (6.8%) | 2,609 (8.8%) | 2,163 (15.5%) |  |
| **Diabetes** | 4,021 (2.7%) | 671 (2.7%) | 1,008 (3.0%) | 1,141 (2.6%) | 784 (2.6%) | 417 (3.0%) | 0.006 |
| **Hypertension** | 35,072 (23.9%) | 5,461 (22.2%) | 7,833 (23.0%) | 10,846 (24.5%) | 7,370 (24.9%) | 3,562 (25.5%) | < 0.001 |
| **Dyslipidemia** | 21,736 (14.8%) | 3,351 (13.6%) | 4,958 (14.5%) | 6,771 (15.3%) | 4,458 (15.0%) | 2,198 (15.7%) | < 0.001 |
| **Glucose (mg/dL)** | 88.5 ± 15.1 | 88.1 ± 14.2 | 88.7 ± 15.7 | 88.7 ± 15.1 | 88.6 ± 14.5 | 88.5 ± 16.4 | < 0.001 |
| **Triglyceride (mg/dL)** | 99.0 ± 39.7 | 98.8 ± 40.2 | 99.0 ± 40.0 | 99.7 ± 39.8 | 98.5 ± 38.7 | 98.0 ± 39.7 | < 0.001 |
| **HDL-C (mg/dL)** | 64.8 ± 14.5 | 64.2 ± 14.8 | 65.1 ± 14.7 | 65.1 ± 14.4 | 64.7 ± 14.3 | 63.9 ± 14.5 | < 0.001 |
| **Creatinine (mg/dL)** | 0.8 ± 0.2 | 0.7 ± 0.2 | 0.8 ± 0.2 | 0.8 ± 0.1 | 0.8 ± 0.1 | 0.8 ± 0.2 | < 0.001 |
| **AST (U/L)** | 23.8 ± 7.4 | 23.5 ± 8.4 | 23.8 ± 7.8 | 23.8 ± 7.1 | 23.8 ± 6.9 | 23.8 ± 6.7 | < 0.001 |
| **ALT (U/L)** | 17.4 ± 7.6 | 17.2 ± 8.0 | 17.5 ± 8.3 | 17.4 ± 7.3 | 17.3 ± 7.1 | 17.3 ± 7.1 | < 0.001 |
| **GGT (U/L)** | 21.4 ± 12.5 | 21.0 ± 13.3 | 21.4 ± 12.2 | 21.4 ± 12.2 | 21.5 ± 12.7 | 21.8 ± 12.8 | < 0.001 |
| **Albumin, (g/dL)** | 4.5 ± 0.3 | 4.5 ± 0.3 | 4.5 ± 0.3 | 4.5 ± 0.3 | 4.5 ± 0.3 | 4.5 ± 0.3 | < 0.001 |
| **Platelet (10⁹/L)** | 254.8 ± 58.4 | 256.6 ± 59.5 | 253.5 ± 57.7 | 253.6 ± 57.4 | 255.1 ± 58.3 | 257.9 ± 60.9 | < 0.001 |
| **Total bilirubin (mg/dL)** | 0.5 ± 0.3 | 0.5 ± 0.3 | 0.5 ± 0.3 | 0.5 ± 0.3 | 0.5 ± 0.3 | 0.5 ± 0.3 | < 0.001 |
| **Tea intake (cups/day)** | 3.4 ± 2.8 | 0.0 ± 0.0 | 1.6 ± 0.5 | 3.5 ± 0.5 | 5.4 ± 0.5 | 9.1 ± 3.4 | - |
| ^1^Mean ± SD; n (%) | | | | | | | |
| ^2^One-way analysis of means (not assuming equal variances); Pearson's Chi-squared test | | | | | | | |
| Values are mean ± SD for continuous variables and n (%) for categorical variables. *P* values are from one-way ANOVA for continuous variables and chi-squared test for categorical variables across tea consumption categories. BMI, body mass index; AST, aspartate aminotransferase; ALT, alanine aminotransferase; GGT, gamma-glutamyl transferase; HDL-C, high-density lipoprotein cholesterol. | | | | | | | |

**Supplementary Table 3. Baseline characteristics of the MetALD cohort by tea consumption category.**

| **Characteristic** | **Overall**  N = 44,748^1^ | **None**  N = 8,649^1^ | **1-2 cups**  N = 12,057^1^ | **3-4 cups**  N = 13,020^1^ | **5-6 cups**  N = 7,694^1^ | **≥ 7 cups**  N = 3,328^1^ | ***P* value**^2^ |
| --- | --- | --- | --- | --- | --- | --- | --- |
| **Age, years** | 58.3 ± 7.5 | 57.4 ± 7.7 | 58.3 ± 7.6 | 58.9 ± 7.3 | 58.3 ± 7.3 | 57.6 ± 7.4 | < 0.001 |
| **Male** | 31,570 (70.6%) | 5,919 (68.4%) | 8,657 (71.8%) | 9,054 (69.5%) | 5,487 (71.3%) | 2,453 (73.7%) | < 0.001 |
| **BMI (kg/m²)** | 28.4 ± 3.6 | 28.9 ± 3.9 | 28.4 ± 3.6 | 28.3 ± 3.5 | 28.3 ± 3.5 | 28.5 ± 3.6 | < 0.001 |
| **Waist circumference (cm)** | 96.6 ± 9.7 | 97.4 ± 10.2 | 96.7 ± 9.7 | 96.1 ± 9.5 | 96.2 ± 9.5 | 97.0 ± 9.8 | < 0.001 |
| **Smoking status** |  |  |  |  |  |  | < 0.001 |
| Never | 17,665 (39.5%) | 3,332 (38.5%) | 4,830 (40.1%) | 5,213 (40.0%) | 3,067 (39.9%) | 1,223 (36.7%) |  |
| Previous | 20,964 (46.8%) | 3,931 (45.5%) | 5,663 (47.0%) | 6,249 (48.0%) | 3,591 (46.7%) | 1,530 (46.0%) |  |
| Current | 6,119 (13.7%) | 1,386 (16.0%) | 1,564 (13.0%) | 1,558 (12.0%) | 1,036 (13.5%) | 575 (17.3%) |  |
| **Diabetes** | 4,448 (9.9%) | 960 (11.1%) | 1,216 (10.1%) | 1,224 (9.4%) | 711 (9.2%) | 337 (10.1%) | < 0.001 |
| **Hypertension** | 22,842 (51.0%) | 4,363 (50.4%) | 6,152 (51.0%) | 6,667 (51.2%) | 3,926 (51.0%) | 1,734 (52.1%) | 0.579 |
| **Dyslipidemia** | 15,326 (34.2%) | 2,986 (34.5%) | 4,221 (35.0%) | 4,405 (33.8%) | 2,579 (33.5%) | 1,135 (34.1%) | 0.182 |
| **Glucose (mg/dL)** | 93.6 ± 21.2 | 94.5 ± 23.0 | 93.8 ± 20.9 | 93.0 ± 19.7 | 93.6 ± 21.6 | 93.0 ± 22.0 | < 0.001 |
| **Triglyceride (mg/dL)** | 182.4 ± 97.4 | 185.0 ± 102.3 | 185.0 ± 98.8 | 180.0 ± 94.7 | 180.4 ± 95.7 | 179.6 ± 93.8 | < 0.001 |
| **HDL-C (mg/dL)** | 55.7 ± 13.5 | 55.8 ± 13.9 | 55.8 ± 13.6 | 55.9 ± 13.5 | 55.4 ± 13.3 | 55.2 ± 13.1 | 0.015 |
| **Creatinine (mg/dL)** | 0.9 ± 0.2 | 0.8 ± 0.2 | 0.9 ± 0.2 | 0.9 ± 0.2 | 0.9 ± 0.2 | 0.9 ± 0.2 | < 0.001 |
| **AST (U/L)** | 28.5 ± 12.7 | 29.1 ± 15.3 | 28.7 ± 13.0 | 28.3 ± 11.0 | 28.0 ± 10.6 | 28.3 ± 13.9 | < 0.001 |
| **ALT (U/L)** | 27.8 ± 16.2 | 28.6 ± 17.8 | 28.0 ± 16.8 | 27.3 ± 14.8 | 27.2 ± 15.4 | 27.5 ± 16.2 | < 0.001 |
| **GGT (U/L)** | 54.6 ± 56.9 | 57.2 ± 60.4 | 55.4 ± 59.6 | 53.5 ± 57.2 | 52.6 ± 50.2 | 53.7 ± 51.0 | < 0.001 |
| **Albumin, (g/dL)** | 4.5 ± 0.3 | 4.5 ± 0.3 | 4.5 ± 0.3 | 4.5 ± 0.3 | 4.5 ± 0.3 | 4.5 ± 0.3 | < 0.001 |
| **Platelet (10⁹/L)** | 247.8 ± 57.2 | 249.1 ± 56.9 | 246.7 ± 56.6 | 246.6 ± 56.9 | 249.3 ± 59.2 | 250.0 ± 57.1 | < 0.001 |
| **Total bilirubin (mg/dL)** | 0.6 ± 0.2 | 0.6 ± 0.2 | 0.6 ± 0.3 | 0.6 ± 0.2 | 0.6 ± 0.2 | 0.5 ± 0.2 | < 0.001 |
| **Tea intake (cups/day)** | 3.1 ± 2.7 | 0.0 ± 0.0 | 1.6 ± 0.5 | 3.5 ± 0.5 | 5.4 ± 0.5 | 9.3 ± 3.5 | - |
| ^1^Mean ± SD; n (%) | | | | | | | |
| ^2^One-way analysis of means (not assuming equal variances); Pearson's Chi-squared test | | | | | | | |
| Values are mean ± SD for continuous variables and n (%) for categorical variables. *P* values are from one-way ANOVA for continuous variables and chi-squared test for categorical variables across tea consumption categories. BMI, body mass index; AST, aspartate aminotransferase; ALT, alanine aminotransferase; GGT, gamma-glutamyl transferase; HDL-C, high-density lipoprotein cholesterol. | | | | | | | |

**Supplementary Table 4. Cox proportional hazards models for tea consumption categories and clinical outcomes in the total cohort.**

| **Outcome** | **N events / N** | **Form** | **Model 1** | **Model 2** | **Model 3** | **Model 4** |
| --- | --- | --- | --- | --- | --- | --- |
| **Death** | 31,797 / 360,548 | None (ref) | 1.00 (ref) | 1.00 (ref) | 1.00 (ref) | 1.00 (ref) |
|  |  | 1-2 vs None | 0.86 (0.83–0.89) *P* < 0.001 | 0.78 (0.75–0.81) *P* < 0.001 | 0.83 (0.80–0.86) *P* < 0.001 | 0.84 (0.81–0.87) *P* < 0.001 |
|  |  | 3-4 vs None | 0.91 (0.88–0.94) *P* < 0.001 | 0.77 (0.74–0.79) *P* < 0.001 | 0.83 (0.80–0.86) *P* < 0.001 | 0.83 (0.81–0.86) *P* < 0.001 |
|  |  | 5-6 vs None | 0.93 (0.90–0.97) *P* < 0.001 | 0.80 (0.77–0.83) *P* < 0.001 | 0.85 (0.82–0.88) *P* < 0.001 | 0.85 (0.82–0.89) *P* < 0.001 |
|  |  | ≥ 7 vs None | 1.11 (1.06–1.15) *P* < 0.001 | 0.98 (0.94–1.02) *P* = 0.315 | 0.98 (0.94–1.02) *P* = 0.308 | 0.96 (0.92–1.01) *P* = 0.095 |
| **Cardiovascular disease** | 29,944 / 360,544 | None (ref) | 1.00 (ref) | 1.00 (ref) | 1.00 (ref) | 1.00 (ref) |
|  |  | 1-2 vs None | 0.95 (0.91–0.98) *P* = 0.003 | 0.87 (0.84–0.91) *P* < 0.001 | 0.92 (0.89–0.96) *P* < 0.001 | 0.94 (0.90–0.97) *P* < 0.001 |
|  |  | 3-4 vs None | 1.00 (0.97–1.04) *P* = 0.822 | 0.89 (0.86–0.92) *P* < 0.001 | 0.96 (0.93–1.00) *P* = 0.042 | 0.98 (0.94–1.01) *P* = 0.209 |
|  |  | 5-6 vs None | 1.02 (0.99–1.06) *P* = 0.193 | 0.91 (0.88–0.95) *P* < 0.001 | 0.97 (0.94–1.01) *P* = 0.129 | 0.98 (0.94–1.02) *P* = 0.236 |
|  |  | ≥ 7 vs None | 1.17 (1.12–1.22) *P* < 0.001 | 1.05 (1.00–1.10) *P* = 0.030 | 1.08 (1.04–1.13) *P* < 0.001 | 1.09 (1.04–1.14) *P* < 0.001 |
| **Cerebrovascular disease** | 13,307 / 360,548 | None (ref) | 1.00 (ref) | 1.00 (ref) | 1.00 (ref) | 1.00 (ref) |
|  |  | 1-2 vs None | 0.88 (0.84–0.93) *P* < 0.001 | 0.81 (0.76–0.85) *P* < 0.001 | 0.84 (0.79–0.89) *P* < 0.001 | 0.83 (0.78–0.88) *P* < 0.001 |
|  |  | 3-4 vs None | 0.95 (0.90–1.00) *P* = 0.054 | 0.80 (0.76–0.85) *P* < 0.001 | 0.85 (0.81–0.90) *P* < 0.001 | 0.84 (0.80–0.89) *P* < 0.001 |
|  |  | 5-6 vs None | 0.96 (0.91–1.01) *P* = 0.120 | 0.82 (0.78–0.87) *P* < 0.001 | 0.86 (0.81–0.91) *P* < 0.001 | 0.84 (0.79–0.89) *P* < 0.001 |
|  |  | ≥ 7 vs None | 1.12 (1.05–1.20) *P* < 0.001 | 1.00 (0.93–1.06) *P* = 0.925 | 1.00 (0.94–1.07) *P* = 0.938 | 0.98 (0.91–1.05) *P* = 0.508 |
| **Chronic kidney disease** | 14,913 / 357,426 | None (ref) | 1.00 (ref) | 1.00 (ref) | 1.00 (ref) | 1.00 (ref) |
|  |  | 1-2 vs None | 0.95 (0.90–1.00) *P* = 0.054 | 0.87 (0.83–0.92) *P* < 0.001 | 0.94 (0.89–0.99) *P* = 0.020 | 0.96 (0.91–1.02) *P* = 0.206 |
|  |  | 3-4 vs None | 1.11 (1.06–1.16) *P* < 0.001 | 0.94 (0.89–0.99) *P* = 0.012 | 1.03 (0.98–1.08) *P* = 0.204 | 1.03 (0.98–1.09) *P* = 0.258 |
|  |  | 5-6 vs None | 1.15 (1.09–1.21) *P* < 0.001 | 1.00 (0.95–1.05) *P* = 0.930 | 1.08 (1.02–1.14) *P* = 0.005 | 1.06 (1.00–1.12) *P* = 0.044 |
|  |  | ≥ 7 vs None | 1.18 (1.11–1.26) *P* < 0.001 | 1.06 (0.99–1.13) *P* = 0.077 | 1.12 (1.05–1.19) *P* < 0.001 | 1.14 (1.06–1.22) *P* < 0.001 |
| Hazard ratios (HR) with 95% confidence intervals (CI) from Cox proportional hazards models comparing tea consumption categories (None = reference). Model 1: crude. Model 2: adjusted for age and sex. Model 3: Model 2 + BMI, diabetes, hypertension, dyslipidemia, and smoking status. Model 4: Model 3 + AST, ALT, GGT, platelet count, albumin, and creatinine. BMI, body mass index; AST, aspartate aminotransferase; ALT, alanine aminotransferase; GGT, gamma-glutamyl transferase. | | | | | | |

**Supplementary Table 5. Cox proportional hazards models for tea consumption categories and clinical outcomes in the no SLD cohort.**

| **Outcome** | **N events / N** | **Form** | **Model 1** | **Model 2** | **Model 3** | **Model 4** |
| --- | --- | --- | --- | --- | --- | --- |
| **Death** | 9,265 / 146,559 | None (ref) | 1.00 (ref) | 1.00 (ref) | 1.00 (ref) | 1.00 (ref) |
|  |  | 1-2 vs None | 0.84 (0.78–0.89) *P* < 0.001 | 0.75 (0.71–0.81) *P* < 0.001 | 0.81 (0.76–0.87) *P* < 0.001 | 0.83 (0.77–0.89) *P* < 0.001 |
|  |  | 3-4 vs None | 0.89 (0.84–0.94) *P* < 0.001 | 0.74 (0.70–0.79) *P* < 0.001 | 0.82 (0.77–0.87) *P* < 0.001 | 0.83 (0.78–0.89) *P* < 0.001 |
|  |  | 5-6 vs None | 0.95 (0.89–1.01) *P* = 0.115 | 0.81 (0.76–0.86) *P* < 0.001 | 0.87 (0.81–0.93) *P* < 0.001 | 0.87 (0.81–0.93) *P* < 0.001 |
|  |  | **≥** 7 vs None | 1.16 (1.08–1.26) *P* < 0.001 | 1.01 (0.93–1.09) *P* = 0.842 | 0.98 (0.91–1.06) *P* = 0.609 | 0.97 (0.89–1.05) *P* = 0.448 |
| **Cardiovascular disease** | 6,743 / 146,558 | None (ref) | 1.00 (ref) | 1.00 (ref) | 1.00 (ref) | 1.00 (ref) |
|  |  | 1-2 vs None | 0.95 (0.88–1.03) *P* = 0.183 | 0.87 (0.80–0.94) *P* < 0.001 | 0.90 (0.83–0.97) *P* = 0.009 | 0.92 (0.85–1.00) *P* = 0.056 |
|  |  | 3-4 vs None | 1.05 (0.97–1.13) *P* = 0.231 | 0.90 (0.83–0.97) *P* = 0.004 | 0.95 (0.89–1.03) *P* = 0.209 | 0.98 (0.91–1.06) *P* = 0.621 |
|  |  | 5-6 vs None | 1.05 (0.97–1.14) *P* = 0.188 | 0.92 (0.85–1.00) *P* = 0.039 | 0.94 (0.87–1.02) *P* = 0.141 | 0.95 (0.87–1.03) *P* = 0.206 |
|  |  | **≥** 7 vs None | 1.30 (1.19–1.43) *P* < 0.001 | 1.13 (1.04–1.24) *P* = 0.007 | 1.08 (0.99–1.19) *P* = 0.086 | 1.09 (0.99–1.20) *P* = 0.092 |
| **Cerebrovascular disease** | 3,876 / 146,559 | None (ref) | 1.00 (ref) | 1.00 (ref) | 1.00 (ref) | 1.00 (ref) |
|  |  | 1-2 vs None | 0.85 (0.77–0.94) *P* = 0.002 | 0.77 (0.69–0.85) *P* < 0.001 | 0.80 (0.72–0.88) *P* < 0.001 | 0.81 (0.72–0.90) *P* < 0.001 |
|  |  | 3-4 vs None | 0.94 (0.86–1.04) *P* = 0.212 | 0.78 (0.71–0.86) *P* < 0.001 | 0.82 (0.75–0.91) *P* < 0.001 | 0.81 (0.73–0.90) *P* < 0.001 |
|  |  | 5-6 vs None | 0.99 (0.90–1.10) *P* = 0.910 | 0.84 (0.76–0.93) *P* = 0.001 | 0.86 (0.78–0.96) *P* = 0.005 | 0.85 (0.76–0.95) *P* = 0.004 |
|  |  | **≥** 7 vs None | 1.22 (1.08–1.37) *P* < 0.001 | 1.05 (0.94–1.19) *P* = 0.390 | 1.02 (0.90–1.14) *P* = 0.793 | 0.99 (0.87–1.12) *P* = 0.818 |
| **Chronic kidney disease** | 3,616 / 145,764 | None (ref) | 1.00 (ref) | 1.00 (ref) | 1.00 (ref) | 1.00 (ref) |
|  |  | 1-2 vs None | 0.97 (0.86–1.08) *P* = 0.542 | 0.89 (0.79–1.00) *P* = 0.042 | 0.91 (0.82–1.02) *P* = 0.121 | 0.93 (0.82–1.05) *P* = 0.213 |
|  |  | 3-4 vs None | 1.20 (1.08–1.33) *P* < 0.001 | 1.02 (0.92–1.14) *P* = 0.640 | 1.06 (0.96–1.18) *P* = 0.256 | 1.04 (0.93–1.16) *P* = 0.522 |
|  |  | 5-6 vs None | 1.31 (1.18–1.46) *P* < 0.001 | 1.14 (1.02–1.27) *P* = 0.019 | 1.14 (1.02–1.27) *P* = 0.018 | 1.12 (1.00–1.26) *P* = 0.051 |
|  |  | **≥** 7 vs None | 1.37 (1.20–1.56) *P* < 0.001 | 1.22 (1.07–1.39) *P* = 0.002 | 1.18 (1.04–1.34) *P* = 0.013 | 1.17 (1.02–1.34) *P* = 0.030 |
| Hazard ratios (HR) with 95% confidence intervals (CI) from Cox proportional hazards models comparing tea consumption categories (None = reference). Model 1: crude. Model 2: adjusted for age and sex. Model 3: Model 2 + BMI, diabetes, hypertension, dyslipidemia, and smoking status. Model 4: Model 3 + AST, ALT, GGT, platelet count, albumin, and creatinine. BMI, body mass index; AST, aspartate aminotransferase; ALT, alanine aminotransferase; GGT, gamma-glutamyl transferase. | | | | | | |

**Supplementary Table 6. Cox proportional hazards models for tea consumption categories and clinical outcomes in the MetALD cohort.**

| **Outcome** | **N events / N** | **Form** | **Model 1** | **Model 2** | **Model 3** | **Model 4** |
| --- | --- | --- | --- | --- | --- | --- |
| **Death** | 5,921 / 44,748 | None (ref) | 1.00 (ref) | 1.00 (ref) | 1.00 (ref) | 1.00 (ref) |
|  |  | 1-2 vs None | 0.87 (0.81–0.94) *P* < 0.001 | 0.79 (0.73–0.85) *P* < 0.001 | 0.82 (0.76–0.88) *P* < 0.001 | 0.83 (0.77–0.90) *P* < 0.001 |
|  |  | 3-4 vs None | 0.84 (0.78–0.90) *P* < 0.001 | 0.73 (0.68–0.79) *P* < 0.001 | 0.77 (0.71–0.83) *P* < 0.001 | 0.77 (0.71–0.83) *P* < 0.001 |
|  |  | 5-6 vs None | 0.85 (0.78–0.92) *P* < 0.001 | 0.78 (0.72–0.85) *P* < 0.001 | 0.81 (0.75–0.88) *P* < 0.001 | 0.84 (0.77–0.91) *P* < 0.001 |
|  |  | **≥** 7 vs None | 0.90 (0.81–1.00) *P* = 0.048 | 0.86 (0.77–0.96) *P* = 0.006 | 0.87 (0.78–0.97) *P* = 0.009 | 0.86 (0.76–0.96) *P* = 0.008 |
| **Cardiovascular disease** | 5,197 / 44,748 | None (ref) | 1.00 (ref) | 1.00 (ref) | 1.00 (ref) | 1.00 (ref) |
|  |  | 1-2 vs None | 1.00 (0.92–1.08) *P* = 0.957 | 0.92 (0.85–1.00) *P* = 0.059 | 0.95 (0.88–1.04) *P* = 0.258 | 0.96 (0.88–1.04) *P* = 0.329 |
|  |  | 3-4 vs None | 0.99 (0.92–1.08) *P* = 0.887 | 0.91 (0.84–0.98) *P* = 0.016 | 0.97 (0.89–1.05) *P* = 0.414 | 0.97 (0.89–1.06) *P* = 0.526 |
|  |  | 5-6 vs None | 1.01 (0.93–1.11) *P* = 0.754 | 0.95 (0.87–1.04) *P* = 0.264 | 0.99 (0.91–1.09) *P* = 0.871 | 1.00 (0.90–1.10) *P* = 0.919 |
|  |  | **≥** 7 vs None | 1.09 (0.97–1.22) *P* = 0.129 | 1.04 (0.93–1.16) *P* = 0.521 | 1.04 (0.93–1.17) *P* = 0.453 | 1.05 (0.93–1.19) *P* = 0.416 |
| **Cerebrovascular disease** | 2,325 / 44,748 | None (ref) | 1.00 (ref) | 1.00 (ref) | 1.00 (ref) | 1.00 (ref) |
|  |  | 1-2 vs None | 0.95 (0.85–1.07) *P* = 0.434 | 0.86 (0.77–0.97) *P* = 0.017 | 0.88 (0.78–0.99) *P* = 0.040 | 0.87 (0.76–0.98) *P* = 0.025 |
|  |  | 3-4 vs None | 0.88 (0.78–0.99) *P* = 0.041 | 0.77 (0.69–0.87) *P* < 0.001 | 0.81 (0.72–0.91) *P* < 0.001 | 0.82 (0.72–0.93) *P* = 0.002 |
|  |  | 5-6 vs None | 0.94 (0.82–1.07) *P* = 0.346 | 0.86 (0.76–0.99) *P* = 0.031 | 0.88 (0.77–1.01) *P* = 0.066 | 0.86 (0.75–0.99) *P* = 0.042 |
|  |  | **≥** 7 vs None | 0.95 (0.80–1.13) *P* = 0.577 | 0.92 (0.77–1.10) *P* = 0.352 | 0.91 (0.77–1.09) *P* = 0.315 | 0.89 (0.74–1.08) *P* = 0.237 |
| **Chronic kidney disease** | 2,165 / 44,297 | None (ref) | 1.00 (ref) | 1.00 (ref) | 1.00 (ref) | 1.00 (ref) |
|  |  | 1-2 vs None | 0.97 (0.86–1.10) *P* = 0.665 | 0.88 (0.78–1.00) *P* = 0.055 | 0.93 (0.82–1.05) *P* = 0.248 | 0.97 (0.84–1.11) *P* = 0.627 |
|  |  | 3-4 vs None | 1.09 (0.96–1.23) *P* = 0.185 | 0.94 (0.83–1.07) *P* = 0.348 | 1.02 (0.90–1.15) *P* = 0.798 | 0.96 (0.84–1.10) *P* = 0.554 |
|  |  | 5-6 vs None | 1.00 (0.87–1.15) *P* = 0.989 | 0.93 (0.80–1.07) *P* = 0.291 | 0.99 (0.86–1.14) *P* = 0.845 | 0.98 (0.84–1.14) *P* = 0.775 |
|  |  | **≥** 7 vs None | 1.02 (0.85–1.22) *P* = 0.850 | 1.01 (0.84–1.21) *P* = 0.928 | 1.03 (0.86–1.24) *P* = 0.714 | 1.04 (0.85–1.26) *P* = 0.716 |
| Hazard ratios (HR) with 95% confidence intervals (CI) from Cox proportional hazards models comparing tea consumption categories (None = reference). Model 1: crude. Model 2: adjusted for age and sex. Model 3: Model 2 + BMI, diabetes, hypertension, dyslipidemia, and smoking status. Model 4: Model 3 + AST, ALT, GGT, platelet count, albumin, and creatinine. BMI, body mass index; AST, aspartate aminotransferase; ALT, alanine aminotransferase; GGT, gamma-glutamyl transferase. | | | | | | |

**Supplementary Table 7. IPTW-weighted baseline characteristics by tea consumption category in the total cohort (N = 360,548), with SMDs before and after weighting.**

| **Variable** | **None** | **1-2 cups** | **3-4 cups** | **5-6 cups** | **≥7 cups** | **SMD (unweighted)** | **SMD (weighted)** |
| --- | --- | --- | --- | --- | --- | --- | --- |
| **Age, years** | 55.8 ± 8.1 | 55.9 ± 8.2 | 55.8 ± 8.2 | 55.9 ± 8.0 | 55.8 ± 7.9 | 0.214 | 0.010 |
| **Male sex, %** | 45.7 | 46.1 | 46.0 | 46.0 | 46.2 | 0.077 | 0.010 |
| **BMI, kg/m²** | 27.0 ± 4.7 | 27.0 ± 4.6 | 27.0 ± 4.6 | 27.0 ± 4.5 | 27.1 ± 4.5 | 0.160 | 0.010 |
| **Current smoker, %** | 10.5 | 10.2 | 10.2 | 10.2 | 10.5 | 0.241 | 0.010 |
| **Diabetes, %** | 8.8 | 8.6 | 8.6 | 8.6 | 8.5 | 0.052 | 0.009 |
| **Hypertension, %** | 38.3 | 38.2 | 38.2 | 38.2 | 38.4 | 0.031 | 0.005 |
| **Dyslipidemia, %** | 25.3 | 25.3 | 25.2 | 25.4 | 25.2 | 0.012 | 0.005 |
| **AST, U/L** | 25.8 ± 9.0 | 25.8 ± 8.9 | 25.8 ± 8.9 | 25.8 ± 8.6 | 25.8 ± 8.1 | 0.061 | 0.003 |
| **ALT, U/L** | 23.0 ± 13.4 | 23.0 ± 13.5 | 23.0 ± 13.1 | 23.0 ± 12.8 | 23.0 ± 13.2 | 0.062 | 0.004 |
| **GGT, U/L** | 35.2 ± 35.8 | 35.2 ± 36.3 | 35.2 ± 36.9 | 35.2 ± 36.0 | 35.3 ± 34.7 | 0.042 | 0.003 |
| **Platelet, 10⁹/L** | 253.8 ± 59.4 | 253.7 ± 59.9 | 253.7 ± 59.0 | 253.6 ± 59.0 | 253.7 ± 59.8 | 0.079 | 0.003 |
| **Albumin, g/dL** | 4.5 ± 0.3 | 4.5 ± 0.3 | 4.5 ± 0.3 | 4.5 ± 0.3 | 4.5 ± 0.3 | 0.121 | 0.003 |
| **Creatinine, mg/dL** | 0.8 ± 0.2 | 0.8 ± 0.2 | 0.8 ± 0.2 | 0.8 ± 0.2 | 0.8 ± 0.2 | 0.139 | 0.007 |
| Cells: IPTW-weighted mean ± SD (continuous) or weighted % (categorical). SMD = maximum pairwise standardized mean difference across the 5 tea consumption categories; SMD < 0.10 indicates adequate balance. Propensity model: tea consumption category regressed on age, sex, BMI, diabetes, hypertension, dyslipidemia, current smoker, AST, ALT, GGT, platelet, albumin, and log-creatinine; stabilized multinomial-GLM weights trimmed at 1st/99th percentiles. ALT, alanine aminotransferase; AST, aspartate aminotransferase; BMI, body mass index; GGT, gamma-glutamyl transferase; GLM, generalized linear model; IPTW, inverse probability of treatment weighting; SD, standard deviation; SMD, standardized mean difference. | | | | | | | |

**Supplementary Table 8. IPTW-weighted baseline characteristics by tea consumption category in the No SLD cohort (N = 146,559), with SMDs before and after weighting.**

| **Variable** | **None** | **1-2 cups** | **3-4 cups** | **5-6 cups** | **≥7 cups** | **SMD (unweighted)** | **SMD (weighted)** |
| --- | --- | --- | --- | --- | --- | --- | --- |
| **Age, years** | 54.6 ± 8.3 | 54.7 ± 8.4 | 54.7 ± 8.2 | 54.7 ± 8.0 | 54.7 ± 8.0 | 0.208 | 0.007 |
| **Male sex, %** | 22.9 | 23.0 | 22.9 | 23.0 | 22.9 | 0.092 | 0.003 |
| **BMI, kg/m²** | 23.5 ± 2.4 | 23.5 ± 2.3 | 23.5 ± 2.3 | 23.5 ± 2.3 | 23.6 ± 2.4 | 0.083 | 0.012 |
| **Current smoker, %** | 9.5 | 9.4 | 9.3 | 9.4 | 9.8 | 0.294 | 0.017 |
| **Diabetes, %** | 2.7 | 2.8 | 2.7 | 2.8 | 2.8 | 0.025 | 0.004 |
| **Hypertension, %** | 23.9 | 24.0 | 23.9 | 24.0 | 24.0 | 0.091 | 0.002 |
| **Dyslipidemia, %** | 15.0 | 15.1 | 15.1 | 15.1 | 15.1 | 0.067 | 0.004 |
| **AST, U/L** | 23.7 ± 7.8 | 23.7 ± 7.2 | 23.7 ± 7.5 | 23.7 ± 6.4 | 23.8 ± 6.6 | 0.043 | 0.007 |
| **ALT, U/L** | 17.3 ± 8.4 | 17.3 ± 7.8 | 17.3 ± 7.4 | 17.3 ± 6.9 | 17.3 ± 7.2 | 0.046 | 0.006 |
| **GGT, U/L** | 21.4 ± 13.8 | 21.4 ± 12.3 | 21.4 ± 11.9 | 21.4 ± 12.1 | 21.5 ± 12.2 | 0.057 | 0.008 |
| **Platelet, 10⁹/L** | 254.6 ± 59.3 | 254.5 ± 57.4 | 254.6 ± 58.8 | 254.7 ± 57.7 | 254.7 ± 59.5 | 0.065 | 0.003 |
| **Albumin, g/dL** | 4.5 ± 0.3 | 4.5 ± 0.3 | 4.5 ± 0.3 | 4.5 ± 0.3 | 4.5 ± 0.3 | 0.111 | 0.004 |
| **Creatinine, mg/dL** | 0.8 ± 0.2 | 0.8 ± 0.2 | 0.8 ± 0.2 | 0.8 ± 0.1 | 0.8 ± 0.1 | 0.140 | 0.009 |
| Cells: IPTW-weighted mean ± SD (continuous) or weighted % (categorical). SMD = maximum pairwise standardized mean difference across the 5 tea consumption categories; SMD < 0.10 indicates adequate balance. Propensity model: tea consumption category regressed on age, sex, BMI, diabetes, hypertension, dyslipidemia, current smoker, AST, ALT, GGT, platelet, albumin, and log-creatinine; stabilized multinomial-GLM weights trimmed at 1st/99th percentiles. ALT, alanine aminotransferase; AST, aspartate aminotransferase; BMI, body mass index; GGT, gamma-glutamyl transferase; GLM, generalized linear model; IPTW, inverse probability of treatment weighting; SD, standard deviation; SMD, standardized mean difference. | | | | | | | |

**Supplementary Table 9. IPTW-weighted baseline characteristics by tea consumption category in the MASLD cohort (N = 169,241), with SMDs before and after weighting.**

| **Variable** | **None** | **1-2 cups** | **3-4 cups** | **5-6 cups** | **≥7 cups** | **SMD (unweighted)** | **SMD (weighted)** |
| --- | --- | --- | --- | --- | --- | --- | --- |
| **Age, years** | 56.2 ± 8.1 | 56.3 ± 8.2 | 56.2 ± 8.1 | 56.3 ± 7.8 | 56.2 ± 7.8 | 0.257 | 0.013 |
| **Male sex, %** | 57.9 | 58.3 | 58.3 | 58.4 | 58.4 | 0.106 | 0.011 |
| **BMI, kg/m²** | 29.7 ± 4.4 | 29.6 ± 4.4 | 29.6 ± 4.4 | 29.6 ± 4.2 | 29.6 ± 4.3 | 0.213 | 0.022 |
| **Current smoker, %** | 9.9 | 9.7 | 9.7 | 9.7 | 9.8 | 0.227 | 0.008 |
| **Diabetes, %** | 13.3 | 13.0 | 13.0 | 13.1 | 13.0 | 0.064 | 0.008 |
| **Hypertension, %** | 47.5 | 47.4 | 47.3 | 47.4 | 47.3 | 0.061 | 0.003 |
| **Dyslipidemia, %** | 31.8 | 31.7 | 31.6 | 31.8 | 31.7 | 0.029 | 0.004 |
| **AST, U/L** | 26.9 ± 9.9 | 27.0 ± 10.6 | 27.0 ± 9.5 | 27.0 ± 10.2 | 27.0 ± 9.4 | 0.049 | 0.010 |
| **ALT, U/L** | 26.7 ± 15.0 | 26.8 ± 15.6 | 26.8 ± 15.0 | 26.8 ± 15.0 | 26.8 ± 15.7 | 0.036 | 0.008 |
| **GGT, U/L** | 42.1 ± 40.6 | 42.2 ± 40.1 | 42.2 ± 37.9 | 42.2 ± 37.9 | 42.5 ± 36.0 | 0.042 | 0.008 |
| **Platelet, 10⁹/L** | 254.8 ± 60.2 | 254.6 ± 60.6 | 254.7 ± 60.8 | 254.5 ± 60.6 | 254.7 ± 59.6 | 0.057 | 0.006 |
| **Albumin, g/dL** | 4.5 ± 0.3 | 4.5 ± 0.3 | 4.5 ± 0.3 | 4.5 ± 0.3 | 4.5 ± 0.3 | 0.108 | 0.006 |
| **Creatinine, mg/dL** | 0.8 ± 0.2 | 0.8 ± 0.2 | 0.8 ± 0.2 | 0.8 ± 0.2 | 0.8 ± 0.2 | 0.129 | 0.006 |
| Cells: IPTW-weighted mean ± SD (continuous) or weighted % (categorical). SMD = maximum pairwise standardized mean difference across the 5 tea consumption categories; SMD < 0.10 indicates adequate balance. Propensity model: tea consumption category regressed on age, sex, BMI, diabetes, hypertension, dyslipidemia, current smoker, AST, ALT, GGT, platelet, albumin, and log-creatinine; stabilized multinomial-GLM weights trimmed at 1st/99th percentiles. ALT, alanine aminotransferase; AST, aspartate aminotransferase; BMI, body mass index; GGT, gamma-glutamyl transferase; GLM, generalized linear model; IPTW, inverse probability of treatment weighting; SD, standard deviation; SMD, standardized mean difference. | | | | | | | |

**Supplementary Table 10. IPTW-weighted baseline characteristics by tea consumption category in the MetALD cohort (N = 44,748), with SMDs before and after weighting.**

| **Variable** | **None** | **1-2 cups** | **3-4 cups** | **5-6 cups** | **≥7 cups** | **SMD (unweighted)** | **SMD (weighted)** |
| --- | --- | --- | --- | --- | --- | --- | --- |
| **Age, years** | 58.2 ± 7.5 | 58.3 ± 7.6 | 58.3 ± 7.5 | 58.3 ± 7.3 | 58.2 ± 7.2 | 0.207 | 0.008 |
| **Male sex, %** | 70.7 | 71.0 | 70.9 | 71.0 | 71.1 | 0.114 | 0.009 |
| **BMI, kg/m²** | 28.5 ± 3.7 | 28.5 ± 3.7 | 28.4 ± 3.6 | 28.4 ± 3.6 | 28.5 ± 3.6 | 0.172 | 0.018 |
| **Current smoker, %** | 14.0 | 13.7 | 13.7 | 13.6 | 13.8 | 0.156 | 0.010 |
| **Diabetes, %** | 10.2 | 10.0 | 10.0 | 10.1 | 9.9 | 0.060 | 0.011 |
| **Hypertension, %** | 51.2 | 51.1 | 51.1 | 51.1 | 51.1 | 0.035 | 0.003 |
| **Dyslipidemia, %** | 34.7 | 34.5 | 34.5 | 34.5 | 34.3 | 0.028 | 0.009 |
| **AST, U/L** | 28.5 ± 13.7 | 28.5 ± 12.0 | 28.5 ± 11.2 | 28.4 ± 11.1 | 28.5 ± 13.4 | 0.082 | 0.010 |
| **ALT, U/L** | 27.9 ± 16.4 | 27.8 ± 16.4 | 27.8 ± 15.3 | 27.7 ± 15.8 | 27.8 ± 17.5 | 0.089 | 0.009 |
| **GGT, U/L** | 55.1 ± 55.8 | 54.6 ± 57.7 | 54.7 ± 57.8 | 54.5 ± 54.5 | 54.6 ± 53.3 | 0.090 | 0.011 |
| **Platelet, 10⁹/L** | 247.9 ± 56.3 | 247.7 ± 57.3 | 247.7 ± 57.2 | 247.6 ± 58.7 | 248.1 ± 56.2 | 0.059 | 0.010 |
| **Albumin, g/dL** | 4.5 ± 0.3 | 4.5 ± 0.3 | 4.5 ± 0.3 | 4.5 ± 0.3 | 4.5 ± 0.3 | 0.069 | 0.008 |
| **Creatinine, mg/dL** | 0.9 ± 0.2 | 0.9 ± 0.2 | 0.9 ± 0.2 | 0.9 ± 0.2 | 0.9 ± 0.2 | 0.198 | 0.010 |
| Cells: IPTW-weighted mean ± SD (continuous) or weighted % (categorical). SMD = maximum pairwise standardized mean difference across the 5 tea consumption categories; SMD < 0.10 indicates adequate balance. Propensity model: tea consumption category regressed on age, sex, BMI, diabetes, hypertension, dyslipidemia, current smoker, AST, ALT, GGT, platelet, albumin, and log-creatinine; stabilized multinomial-GLM weights trimmed at 1st/99th percentiles. ALT, alanine aminotransferase; AST, aspartate aminotransferase; BMI, body mass index; GGT, gamma-glutamyl transferase; GLM, generalized linear model; IPTW, inverse probability of treatment weighting; SD, standard deviation; SMD, standardized mean difference. | | | | | | | |

**Supplementary Table 11. IPTW-weighted Cox regression for tea consumption category and clinical outcomes across 4 cohorts.**

| **Cohort** | **Outcome** | **None (ref)** | **1-2 vs None** | **3-4 vs None** | **5-6 vs None** | **≥7 vs None** |
| --- | --- | --- | --- | --- | --- | --- |
| Total cohort | Death | 1.00 (ref) | 0.85 (0.78-0.92) *P* < 0.001 | 0.84 (0.78-0.91) *P* < 0.001 | 0.89 (0.81-0.97) *P* = 0.009 | 1.02 (0.92-1.13) *P* = 0.686 |
|  | Cardiovascular disease | 1.00 (ref) | 0.92 (0.84-1.01) *P* = 0.087 | 0.98 (0.90-1.06) *P* = 0.561 | 1.01 (0.92-1.11) *P* = 0.797 | 1.14 (1.02-1.27) *P* = 0.017 |
|  | Cerebrovascular disease | 1.00 (ref) | 0.80 (0.70-0.91) *P* < 0.001 | 0.88 (0.78-1.00) *P* = 0.052 | 0.84 (0.73-0.96) *P* = 0.011 | 0.87 (0.74-1.03) *P* = 0.113 |
|  | Chronic kidney disease | 1.00 (ref) | 0.90 (0.79-1.03) *P* = 0.115 | 0.89 (0.79-1.00) *P* = 0.059 | 0.88 (0.77-1.00) *P* = 0.044 | 0.96 (0.82-1.12) *P* = 0.567 |
| No SLD | Death | 1.00 (ref) | 0.82 (0.74-0.91) *P* < 0.001 | 0.79 (0.72-0.88) *P* < 0.001 | 0.82 (0.74-0.91) *P* < 0.001 | 0.97 (0.86-1.09) *P* = 0.595 |
|  | Cardiovascular disease | 1.00 (ref) | 0.95 (0.84-1.08) *P* = 0.415 | 0.97 (0.86-1.09) *P* = 0.554 | 0.89 (0.78-1.01) *P* = 0.076 | 1.09 (0.95-1.27) *P* = 0.229 |
|  | Cerebrovascular disease | 1.00 (ref) | 0.89 (0.76-1.05) *P* = 0.166 | 0.83 (0.71-0.97) *P* = 0.020 | 0.87 (0.74-1.03) *P* = 0.096 | 1.12 (0.93-1.35) *P* = 0.233 |
|  | Chronic kidney disease | 1.00 (ref) | 0.92 (0.77-1.09) *P* = 0.331 | 0.95 (0.81-1.12) *P* = 0.549 | 0.99 (0.83-1.18) *P* = 0.901 | 1.00 (0.81-1.23) *P* = 0.995 |
| MASLD | Death | 1.00 (ref) | 0.83 (0.76-0.90) *P* < 0.001 | 0.81 (0.75-0.88) *P* < 0.001 | 0.89 (0.82-0.96) *P* = 0.005 | 0.94 (0.85-1.04) *P* = 0.210 |
|  | Cardiovascular disease | 1.00 (ref) | 0.90 (0.83-0.98) *P* = 0.013 | 0.94 (0.87-1.01) *P* = 0.090 | 0.93 (0.86-1.00) *P* = 0.065 | 1.06 (0.96-1.16) *P* = 0.231 |
|  | Cerebrovascular disease | 1.00 (ref) | 0.85 (0.74-0.96) *P* = 0.010 | 0.84 (0.74-0.94) *P* = 0.003 | 0.84 (0.74-0.95) *P* = 0.006 | 0.99 (0.85-1.15) *P* = 0.877 |
|  | Chronic kidney disease | 1.00 (ref) | 0.95 (0.84-1.06) *P* = 0.348 | 0.93 (0.84-1.04) *P* = 0.210 | 0.95 (0.85-1.06) *P* = 0.378 | 0.95 (0.82-1.09) *P* = 0.431 |
| MetALD | Death | 1.00 (ref) | 0.84 (0.77-0.91) *P* < 0.001 | 0.78 (0.72-0.84) *P* < 0.001 | 0.83 (0.76-0.90) *P* < 0.001 | 0.82 (0.73-0.93) *P* = 0.001 |
|  | Cardiovascular disease | 1.00 (ref) | 0.97 (0.89-1.05) *P* = 0.443 | 0.98 (0.90-1.06) *P* = 0.578 | 1.00 (0.91-1.10) *P* = 0.994 | 1.03 (0.91-1.17) *P* = 0.605 |
|  | Cerebrovascular disease | 1.00 (ref) | 0.89 (0.78-1.01) *P* = 0.075 | 0.84 (0.74-0.95) *P* = 0.006 | 0.89 (0.77-1.02) *P* = 0.093 | 0.87 (0.72-1.05) *P* = 0.150 |
|  | Chronic kidney disease | 1.00 (ref) | 0.92 (0.80-1.05) *P* = 0.217 | 0.95 (0.83-1.09) *P* = 0.457 | 0.88 (0.76-1.03) *P* = 0.112 | 0.92 (0.76-1.12) *P* = 0.408 |
| Hazard ratios were estimated by Cox proportional hazards regression with stabilized inverse probability of treatment weights derived from a multinomial generalized linear propensity model regressing tea consumption category (None / 1-2 / 3-4 / 5-6 / ≥7 cups/day) on age, sex, BMI, diabetes, hypertension, dyslipidemia, current smoker, AST, ALT, GGT, platelet, albumin, and log-creatinine (winsorized at the 1st/99th percentiles). Weights were trimmed at the 1st and 99th percentiles. Robust (sandwich) standard errors were used. ALT, alanine aminotransferase; AST, aspartate aminotransferase; CI, confidence interval; GGT, gamma-glutamyl transferase; HR, hazard ratio; IPTW, inverse probability of treatment weighting; SLD, steatotic liver disease; MASLD, metabolic dysfunction-associated steatotic liver disease; MetALD, metabolic dysfunction and alcohol-associated liver disease. | | | | | | |

**Supplementary Table 12. J-shape-aware multi-method validation across 4 cohorts (Cox quadratic, Poisson IRR per 1 cup/day, AFT Weibull TR per 1 cup/day, and Cox HR per 1 cup of distance from the RCS nadir at 2 cups/day).**

| **Cohort** | **Outcome** | **Cox quadratic β2 (P)** | **Cox quadratic nadir** | **Poisson IRR per cup** | **AFT Weibull TR per cup** | **Cox HR per cup distance from optimum** |
| --- | --- | --- | --- | --- | --- | --- |
| Total cohort | Death | β2 = 0.0075 *P* < 0.001 | 4.49 cups/day | 1.001 (0.997–1.005) *P* = 0.615 | 0.999 (0.997–1.002) *P* = 0.548 | 1.025 (1.020–1.031) *P* < 0.001 |
|  | Cardiovascular disease | β2 = 0.0030 *P* < 0.001 | 2.78 cups/day | 1.011 (1.007–1.015) *P* < 0.001 | 0.990 (0.986–0.994) *P* < 0.001 | 1.022 (1.016–1.027) *P* < 0.001 |
|  | Cerebrovascular disease | β2 = 0.0063 *P* < 0.001 | 4.35 cups/day | 1.002 (0.996–1.009) *P* = 0.475 | 0.998 (0.993–1.003) *P* = 0.464 | 1.024 (1.016–1.033) *P* < 0.001 |
|  | Chronic kidney disease | β2 = 0.0004 *P* = 0.590 | -17.32 cups/day | 1.020 (1.014–1.026) *P* < 0.001 | 0.984 (0.979–0.989) *P* < 0.001 | 1.026 (1.018–1.035) *P* < 0.001 |
| No SLD | Death | β2 = 0.0084 *P* < 0.001 | 4.25 cups/day | 1.006 (0.999–1.014) *P* = 0.104 | 0.996 (0.991–1.001) *P* = 0.101 | 1.033 (1.023–1.043) *P* < 0.001 |
|  | Cardiovascular disease | β2 = 0.0041 *P* < 0.001 | 2.97 cups/day | 1.014 (1.005–1.023) *P* = 0.003 | 0.988 (0.980–0.996) *P* = 0.003 | 1.028 (1.016–1.040) *P* < 0.001 |
|  | Cerebrovascular disease | β2 = 0.0084 *P* < 0.001 | 4.05 cups/day | 1.010 (0.998–1.022) *P* = 0.118 | 0.993 (0.984–1.002) *P* = 0.116 | 1.039 (1.023–1.055) *P* < 0.001 |
|  | Chronic kidney disease | β2 = -0.0004 *P* = 0.818 | 41.46 cups/day | 1.029 (1.017–1.042) *P* < 0.001 | 0.975 (0.965–0.986) *P* < 0.001 | 1.037 (1.020–1.053) *P* < 0.001 |
| MASLD | Death | β2 = 0.0063 *P* < 0.001 | 4.14 cups/day | 1.006 (1.000–1.012) *P* = 0.041 | 0.996 (0.993–1.000) *P* = 0.029 | 1.026 (1.019–1.034) *P* < 0.001 |
|  | Cardiovascular disease | β2 = 0.0028 *P* < 0.001 | 2.91 cups/day | 1.009 (1.004–1.015) *P* < 0.001 | 0.991 (0.986–0.996) *P* < 0.001 | 1.019 (1.012–1.027) *P* < 0.001 |
|  | Cerebrovascular disease | β2 = 0.0055 *P* < 0.001 | 4.21 cups/day | 1.004 (0.995–1.013) *P* = 0.373 | 0.997 (0.990–1.004) *P* = 0.354 | 1.023 (1.011–1.034) *P* < 0.001 |
|  | Chronic kidney disease | β2 = 0.0006 *P* = 0.573 | -8.15 cups/day | 1.015 (1.007–1.023) *P* < 0.001 | 0.987 (0.981–0.994) *P* < 0.001 | 1.021 (1.011–1.031) *P* < 0.001 |
| MetALD | Death | β2 = 0.0072 *P* < 0.001 | 5.40 cups/day | 0.984 (0.973–0.994) *P* = 0.002 | 1.010 (1.004–1.017) *P* = 0.002 | 1.012 (0.998–1.026) *P* = 0.100 |
|  | Cardiovascular disease | β2 = 0.0020 *P* = 0.158 | 2.53 cups/day | 1.007 (0.997–1.018) *P* = 0.177 | 0.993 (0.983–1.003) *P* = 0.179 | 1.016 (1.001–1.031) *P* = 0.035 |
|  | Cerebrovascular disease | β2 = 0.0033 *P* = 0.147 | 6.51 cups/day | 0.986 (0.969–1.002) *P* = 0.090 | 1.011 (0.999–1.023) *P* = 0.084 | 1.000 (0.978–1.023) *P* = 0.991 |
|  | Chronic kidney disease | β2 = 0.0015 *P* = 0.503 | 1.10 cups/day | 1.010 (0.993–1.027) *P* = 0.249 | 0.992 (0.979–1.006) *P* = 0.255 | 1.015 (0.992–1.039) *P* = 0.206 |
| The four orthogonal methods test J-shape from different angles. (1) Cox quadratic: linear (β₁) and quadratic (β₂) tea terms; β₂ *P* < 0.05 indicates curvature; nadir = -β₁/(2·β₂). (2) Poisson regression with log(person-years) offset: incidence rate ratio per 1 cup/day. (3) Weibull AFT: time ratio per 1 cup/day (TR < 1 = shortened time-to-event). (4) Cox model with distance-from-optimum (\|tea − 2\|): hazard ratio per 1 cup of deviation from the RCS nadir. Models were adjusted for age, sex, body mass index, smoking, diabetes, hypertension, and dyslipidemia. Tea was winsorized at the 99th percentile. AFT, accelerated failure time; CI, confidence interval; HR, hazard ratio; IRR, incidence rate ratio; RCS, restricted cubic spline; TR, time ratio. | | | | | | |
